# Author Correction: Geophysical evidence for an enriched molten silicate layer above Mars’s core

**DOI:** 10.1038/s41586-023-06930-8

**Published:** 2024-01-02

**Authors:** Henri Samuel, Mélanie Drilleau, Attilio Rivoldini, Zongbo Xu, Quancheng Huang, Raphaël F. Garcia, Vedran Lekić, Jessica C. E. Irving, James Badro, Philippe H. Lognonné, James A. D. Connolly, Taichi Kawamura, Tamara Gudkova, William B. Banerdt

**Affiliations:** 1 Université Paris Cité, Institut de physique du globe de Paris, CNRS, Paris, France; 2 Institut Supérieur de l’Aéronautique et de l’Espace ISAE-SUPAERO, Toulouse, France; 3 Royal Observatory of Belgium, Brussels, Belgium; 4 Department of Geophysics, Colorado School of Mines, Golden, CO USA; 5 University of Maryland, College Park, MD USA; 6 School of Earth Sciences, University of Bristol, Bristol, UK; 7 ETH Zurich, Zurich, Switzerland; 8 Schmidt Institute of Physics of the Earth, Russian Academy of Sciences, Moscow, Russia; 9 Jet Propulsion Laboratory, California Institute of Technology, Pasadena, CA USA

**Keywords:** Seismology, Geophysics, Geodynamics

Correction to: *Nature* 10.1038/s41586-023-06601-8 Published online 25 October 2023

In the version of the article initially published, the histogram in Fig. 2f was plotted incorrectly. The original and corrected Fig. 2f appear as Fig. 1, below. The figure and associated source data have now been updated in the HTML and PDF versions of the article.

**Fig. 1:**
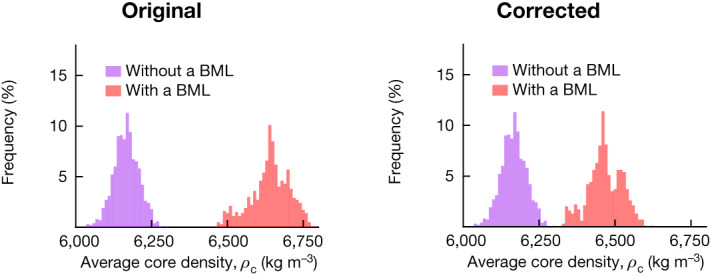
Original and revised Fig. 2f .

