# Supplementary material for: Geophysical evidence for an enriched molten silicate layer above Mars’s core
Source: Nature. 2023 Oct 25;622(7984):712–7. doi: 10.1038/s41586-023-06601-8 (PMC10600000; doi:10.1038/s41586-023-06601-8)
Supplement: Supplementary file 1 — Additional information on the dataset and on inversion and modelling approaches used in this study. Section 1 presents the seismic dataset. Section 2 discusses the existence and the identification of the PbdiffPcP phase. Section 3 provides details on the inversion method. Section 4 displays synthetics seismograms computed from inversion outputs. Section 5 provides information on seismic attenuation constraints from InSight’s seismic data. Section 6 discusses the compatibility of the inversion output models with RISE geodetic data. Section 7 contains information on the core formation modelling used to constrain core composition. Section 8 provides details on the EoS used for the core (Section 8.1) and on the constraints on core composition using BML inversion output models combined with the core EoS (Section 8.2). [file 41586_2023_6601_MOESM1_ESM.pdf]

nature portfolio

https://doi.org/10.1038/s41586-023-06601-8

Supplementary information

Geophysical evidence for an enriched molten silicate layer above Mars's core

In the format provided by the authors and unedited

SUPPLEMENTARY INFORMATION

Geophysical evidence for an enriched molten silicate layer above Mars' core

Henri Samuel, Mélanie Drilleau, Attilio Rivoldini, Zongbo Xu, Quancheng Huang, Raphaël F. Garcia, Vedran Lekić, Jessica C.E. Irving, James Badro, Philippe H. Lognonné, James A. D. Connolly, Taichi Kawamura, Tamara Gudkova, William Bruce Banerdt

Contents

List of Figures 1

1 Seismic travel time data set 2
- 1.1 Picking of PP and P-diffracted phases for event S1000a . . . 4
- 1.2 ScS picking for event S1222a . . . 6

2 Identification of the PbdiffPcP phase 8

3 Inversion approach 9
- 3.1 Note on seismic datafit and inversion results . . . 10

4 Synthetic seismograms 10

5 Q estimation from direct arrivals 11

6 Nutation of BML models 12

7 Core formation modelling 13
- 7.1 Thermodynamic model of element partitioning between metal and silicate . . . 13
- 7.2 Melting of bulk silicate Mars . . . 15
- 7.3 Core composition . . . 16

8 Seismologically constrained core composition 17
- 8.1 Equation of state for the liquid core . . . 18
- 8.2 Seismologically constrained core composition . . . 19

Bibliography 22

List of Figures

1 Analysis of Pdiff (or PbdiffPcP) and PP arrivals of event S1000a. (a) Vertical ground velocity band pass filtered in the 0.4-0.9 Hz range. (b) Spectrogram of vertical ground velocity. (c) Instantaneous phase coherence between different ground velocity components (E=East, N=North and Z=vertical) multiplied by the envelope of vertical or horizontal components in the 0.4-1 Hz frequency range. (d) Estimates of the incidence angles using two different methods relying on coherence and correlations between components. . . . 4

2 Frequency-dependent polarization analysis of S1000a waveforms. (a) Vertical ground velocity band pass filtered in the 0.3-0.8 Hz range, with the second panel's vertical limits revealing the smaller amplitude arrivals before the main PP arrival. (b) Z score of the excess vertically-polarized rectilinear motion, computed from panels d and e integrated across frequencies. (c) Amplitude spectrogram of vertical ground velocity. (d) Vertically-polarized and (e) horizontally-polarized rectilinear motion – defined in supplement of Stähler et al. [2021] – scaled by the square root of the spectral amplitude in each time-frequency tile. The time axis is with respect to the PP pick from Irving et al. [2023], which is, like the PbdiffPcP arrival, marked with a dashed green line. . . . 6

3 Waveform matching (first row) and polarization analysis (third and fourth rows) of event S1222a. In the first row, the radial component data (black) is bandpass filtered between 0.3 and 0.9 Hz, and the red dotted wiggle is the S waveform template. The second row is the spectrum of the radial component data bandpass filtered between 0.01 and 1 Hz. 7
4 Raypaths through Mars for diffracted phases in a representative BML model (displayed in panels (g) to (n) of Fig. 1). Background colours indicate compressional-wave velocities throughout Mars. The white dashed line indicates the path of a wave diffracted at the base of the solid mantle (Pdiff, which on Earth diffracts along the core-mantle boundary). The grey lines highlight a selection of possible paths for PbdiffPcP, whose paths are described in detail below, and which exactly follow the Pdiff path in the mantle. 8
5 Examples of velocity models of the non-BML (a), main BML (b) and supplementary BML (c) inversion sets described in Table 1. (d,e,f) are synthetic seismograms showing the vertical velocity for the three sets of models between 123° and 129° of epicentral distances (close to the epicentral distance of S1000a), considering an explosion located at the surface. (g,h,i) are synthetic seismograms showing the radial velocity for the three sets of models between and epicentral distance ranging between 52° and 58°, considering a marsquake located at 5 km depth generated by a normal fault. The predicted body wave arrival times are displayed with coloured lines. The dashed lines correspond to seismic phases, which are not yet observed in the InSight recordings. The synthetic seismograms are filtered between 0.5 and 1 Hz. 11
6 S-wave spectra for impact event S1094b. The red lines is observed spectra computed with 30-second time window and green crosses are the data used for the fit. The blue curve corresponds to the fit. A peak at 2.4 Hz was masked since this is likely a local effect, which is not taken into account in the model above. In addition to the final spectral shape, contribution from the source time function (orange curve) and the attenuation (yellow curve) are displayed. . . 12
7 (a) Apparent (*i.e.,* liquid) core radius (R_l) as a function of FCN period, (b) apparent core radius as a function of core amplification factor F, and (c) FCN period-core amplification correlation. The gray shaded areas represent 1 σ, 2 σ, and 3 σ uncertainty regions. 13
8 Composition of Mars' core from core formation modelling. (left) Oxygen, (right) carbon concentrations are plotted as a function of sulphur content, which then remains the only free compositional parameter of the problem. Silicon in always below 0.1 wt% and negligible, and hydrogen is unconstrained, but is significantly below 0.15 wt %. The Oxygen composition range is bound by the two following polynomials: $O_{\min} = -3.11676103\ 10^{-4}S^3 + 1.98176935\ 10^{-2}S^2 - 7.18932170\ 10^{-2}S + 1.28917783$ and $O_{\max} = -3.(90147450\ 10^{-4}S^3 + 2.58013870\ 10^{-2}S^2 - 2.27374258\ 10^{-2}S + 3.06338724$, and the Carbon composition range is bound by the saturation value (which depends on S and O concentration, Eq. 11) as an upper bound and 0 as a lower bound. 17
9 Effect of light element concentration x on density (a) and acoustic velocity (b) compared to iron at Mars' CMB conditions (full lines) (P = 22 GPa, T = 2760 K) and core center conditions (dashed lines) (P = 40 GPa, T = 3100 K) 19
10 Core density-velocity histogram for the Mars models of this study. The blue, yellow, and green dots represent interior models that have elastic properties compatible with and Fe-O-S, Fe-O-S-H, and Fe-O-S-C-H alloy, respectively. 20
11 Core composition inferred from the output models of the BML set. Core sulfur, oxygen, carbon, and hydrogen weight fraction assuming a liquid core alloy of Fe-O-S, Fe-O-S-H, and Fe-O-S-C-H. 21

1 Seismic travel time data set

We used the body waves arrival times estimated in Drilleau et al. [2022] for seventeen low-frequency marsquakes. In addition to the direct P and S phases, we included phases reflecting from the surface (PP, PPP, SS, SSS) and reflections from a deep mantle solid/liquid boundary (which was assumed to be the CMB in Stähler et al. [2021] and follow-up studies, hence the label ScS but will be at the bottom of the partially melted layer for BML models). Depth phases resulting from a reflection at the surface of Mars close

to the epicenter of the seismic event (pP, sP, sS) were also included. Body waves arrival times of the three largest seismic events detected so far were added to the pre-existing database of Drilleau et al. [2022]: Two meteorite impacts (S1000a, S1094b, [Posiolova et al., 2022; Kim et al., 2022]) and one marsquake (S1222a, [Kawamura et al., 2022]). We also used core-transiting seismic phase (SKS) recently measured in Irving et al. [2023] from two farside seismic events detected on Mars: A marsquake (S0976a) and impact S1000a. For the latter event, we re-evaluated the differential time between the arrivals of the PP and the Pdiff (or PbdiffPcP in the case of BML models) phases to be 180.7±10 s. Supplementary Table 1 lists the complete travel-time data set we used for our inversions.

In addition to the purely seismic data, we considered geodetic data in the form of the degree-two Love number (with both its real and imaginary parts), the moment of inertia factor and constraints on the mass of Mars [Konopliv et al., 2016, 2020]. Finally, models were required to have crustal thicknesses and density that satisfy constraints from both receiver functions and gravity and topography data [Knapmeyer-Endrun et al., 2021; Wieczorek et al., 2022].

The body wave arrival time data set and the locations of the seismic events deduced from our inversions are summarized in Table 1. The locations of the two meteorite impacts S1000a and S1094b are fixed [Posiolova et al., 2022].

The picking of PP and Pdiff (or PbdiffPcP) arrivals for event S1000a is described in detail in Section 1.1. The Pdiff (or PbdiffPcP) phase appears in the 0.4-1 Hz range as a low energy signal, but significantly above the noise, with a low incidence angle [Posiolova et al., 2022]. The arrival time is picked at phase start when the energy passes above the noise. This results in PP-Pdiff (or PP-PbdiffPcP) differential time of 180 s consistent with t_{PP} -t_{Pdiff} =170 s in Posiolova et al. [2022] and close to the value t_{PP} - t_{Pdiff}=177 s listed in Duran et al. [2022].

1.1 Picking of PP and P-diffracted phases for event S1000a

The pick analysis is performed on the seismic record from the InSight mission [InSight Mars SEIS Data Service, 2019]. The P-diffracted and PP phases of event S1000a event have been described in Fig. S1 in Posiolova et al. [2022] and in Duran et al. [2022]. As shown in Fig 1, the Pdiff (or PbdiffPcP) phase appears in the 0.4-1 Hz range as a low energy signal, yet significantly above the noise, with a low incidence angle, and presents an increase of phase coherence between vertical and horizontal components. The arrival time is picked at phase start when the energy is passing above the noise. A similar analysis is performed on PP arrival for which the pick is more difficult due to signal-generated noise prior the main PP-arrival. Arrival times are estimated to be at 11.35±5 s and 192±5 s respectively for Pdiff and PP after the "EVENT START" time defined by MQS (Mars Quake Service) [InSight Marsquake Service, 2023] to be 2021-09-18T17:58:51.68 UTC. Consequently, the differential time PP-Pdiff is estimated to be 180.65±10s, in close agreement with the estimate in Duran et al. [2022] (177±10s).

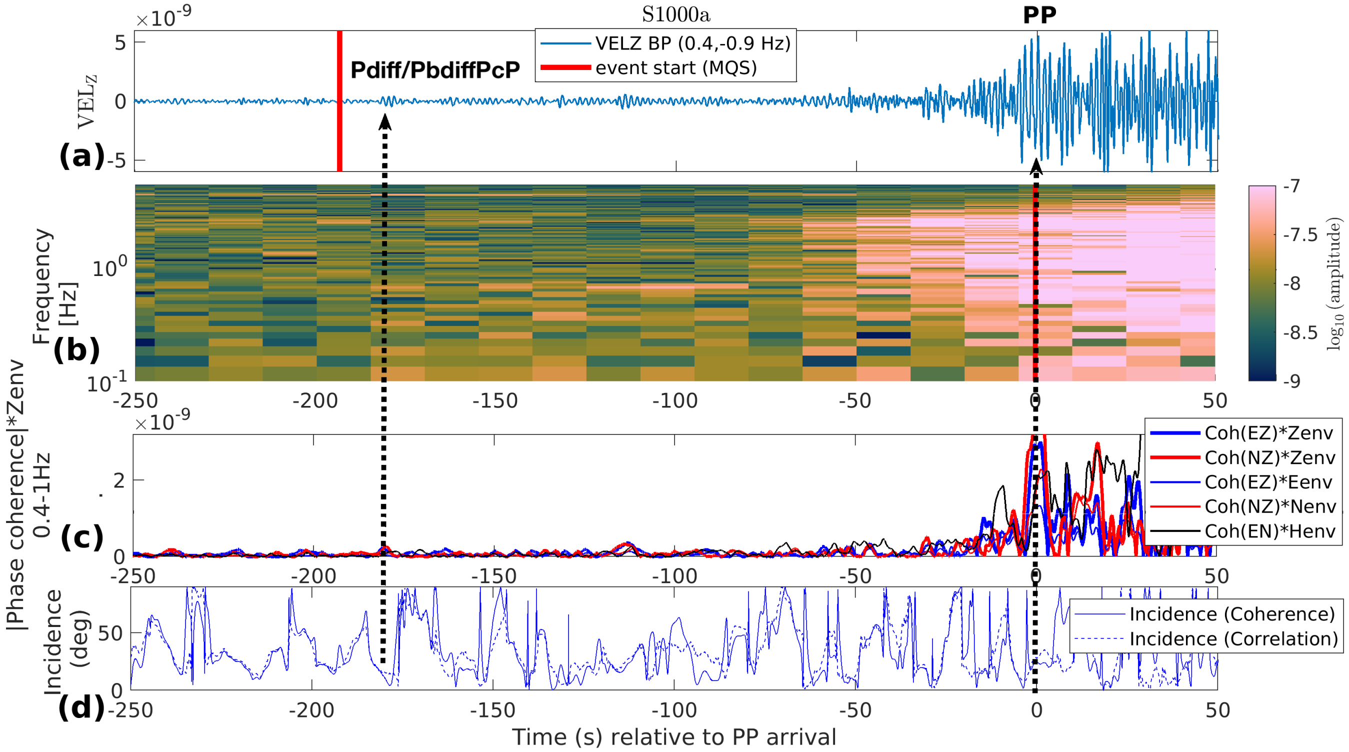

Figure 1: Analysis of Pdiff (or PbdiffPcP) and PP arrivals of event S1000a. (a) Vertical ground velocity band pass filtered in the 0.4-0.9 Hz range. (b) Spectrogram of vertical ground velocity. (c) Instantaneous phase coherence between different ground velocity components (E=East, N=North and Z=vertical) multiplied by the envelope of vertical or horizontal components in the 0.4-1 Hz frequency range. (d) Estimates of the incidence angles using two different methods relying on coherence and correlations between components.

Additional confidence in the pick of P-diffracted and PP phases of event S1000a comes from a frequency-dependent polarization analysis (FDPA) of the three-component waveforms, which is shown in Fig 2. This analysis closely follows that of Method A in Irving et al. [2023], and has successfully been used to identify body waves [*e.g.* Stähler et al., 2021; Irving et al., 2023] as arrivals with a high degree of linear polarization either in the vertical or horizontal directions. In Fig. 2 we modify the approach to weight the polarization attributes in each time-frequency tile by the square root of the amplitude in order to minimize sensitivity to noise. The PbdiffPcP arrival can be seen at 176±10s before the PP arrival and corresponds to a peak in the velocity waveforms (panel a), and excess of vertically-polarized rectilinear motion (σ_{ELVP}, defined as the Z score of the difference between vertically- and horizontally-polarized rectilinear motion, see panel b), a peak in the amplitude spectrum in the 0.55-0.8 Hz frequency range (panel c), and a prominent pulse

Table 1: Summary of observed body-wave differential travel times (in seconds). When the differential time cannot be estimated, "-" marker is indicated. From S0154a to S0918a, the data are taken from Drilleau et al. [2022]. The differential times of S0976a and S1000a are from Irving et al. [2023]. For S1000a, we estimated PP-Pdiff (or PP-PbdiffPcP) = 180.65±10 s. The data of S1094b and S1222a are from [Posiolova et al., 2022] and [Kawamura et al., 2022], respectively. Error bars for direct P- and S- phases arrival times are 5 s. Error bars for PP and SS are 8 s and 5 s, respectively. Error bars for PPP and SSS are 12 s and 8 s, respectively. Concerning the depth phases, the error bars are equal to 3 s for pP arrival times, and 5 s for sP and sS arrival times. Error bars for ScS phases are 12 s. Error bars for SS-PP and SKS-PP are considered to be 10 s. The mean epicentral distance (Δ) and depth of each seismic event constrained by our inversions are also indicated, considering a case with and without a BML.

Event	S-P	pP-P	sP-P	PP-P	PPP-P	sS-S	SS-S	SSS-S	ScS-S	SS-PP	SKS-PP	Δ(°) (no BML)	Depth (no BML)	Δ(°) (BML)	Depth (BML)
S0154a	174.4	-	-	-	-	-	25.3	35	-	-	-	29.9±1.0	22.0±9.6	29.7±2.1	17.4±11.8
S0173a	178.8	-	9.43	19.9	34.4	13.2	24.4	40.5	345.2	-	-	30.9±0.6	20.9±8.4	30.9±1.1	28.4±8.7
S0185a	327.28	4	-	22.47	49.3	10	30.9	55.4	152.3 -	-	-	55.4±0.9	14.4±6.0	54.8±1.2	17.4±11.6
S0235b	171.4	-	-	18.6	32	9.2	23.2	33.3	343.9	-	-	30.1±0.7	21.2±7.4	30.5±1.4	26.1±8.1
S0325a	229.3	9.8	-	21.1	34.4	13.8	26.1	50.3	220.4	-	-	41.9±2.0	30.9±7.7	42.0±2.5	33.8±9.1
S0407a	170.7	6.77	-	23.38	-	13.3	21.1	33.1	370	-	-	28.7±0.8	21.1±5.8	29.1±1.0	31.3±9.4
S0409d	163.2	8.3	-	27.6	36.94	8.4	20.9	39.8	320.1	-	-	29.7±1.7	26.2±8.8	30.6±2.3	26.1±8.7
S0474a	121.6	-	-	13.4	24.8	-	15.8	32.4	-	-	-	20.5±1.2	24.1±9.4	20.7±2.6	30.7±11.6
S0484b	173.1	5.5	-	19.73	-	13	17.4	-	322.3	-	-	28.7±0.8	21.1±5.8	31.3±1.8	24.9±7.6
S0784a	179.3	6.5	-	13.7	22.4	7.2	19.6	28	-	-	-	30.0±1.5	12.9±7.6	30.2±2.6	16.8±11.1
S0802a	180.3	4	-	25.6	33.9	9.3	22.4	36.5	387.6	-	-	28.9±1.7	15.8±7.7	30.0±1.8	20.4±9.1
S0809a	191.95	4.5	-	16.25	29.65	8.1	23.8	39.3	373.5	-	-	30.6±1.7	12.1±9.3	30.7±1.9	16.0±9.6
S0820a	174.1	-	-	21.9	32.1	8.5	-	-	-	-	-	30.1±1.0	15.4±12.0	28.1±3.6	18.7±11.6
S0861a	319.3	-	-	19.6	47.6	-	41.1	-	-	-	-	55.3±1.0	34.3±10.7	54.5±2.6	15.5±12.4
S0864a	171.4	-	-	18	27.9	17.3	26.4	-	-	-	-	29.9±1.4	23.9±8.6	29.0±2.2	25.0±8.9
S0916d	170.8	3.9	-	19.3	36.1	-	19	42.9	342.8	-	-	30.2±0.7	11.1±13.1	30.2±1.5	16.3±10.7
S0918a	102.4	-	-	12.8	22.5	-	21.2	35	-	-	-	16.9±1.0	16.9±7.1	16.6±2.2	22.3±8.3
S0976a	-	-	-	-	-	-	-	-	-	854.4	303.9	143.6±2.0	13.5±12.9	144.0±5.3	30.0±10.8
S1000a	-		-	-	-	-	-	-	-	749.0	339.3	125.9	0.0	125.9	0.0
S1094b	343.0	-	-	-	-	-	-	-	-	-	-	58.5	0.0	58.5	0.0
S1222a	216.0	-	-	-	-	-	-	-	258	-	-	37.3±1.0	14.9±14.3	36.1±2.6	32.8±11.2

of vertically-polarized rectilinear motion in the 0.6-0.8 Hz frequency range (VRM, panel d) without an associated increase in horizontally-polarized rectilinear motion (HRM, panel e). The arrival identified as PbdiffPcP by the FDPA is in agreement with the complementary coherence and incidence angle analysis shown in Fig. 1.

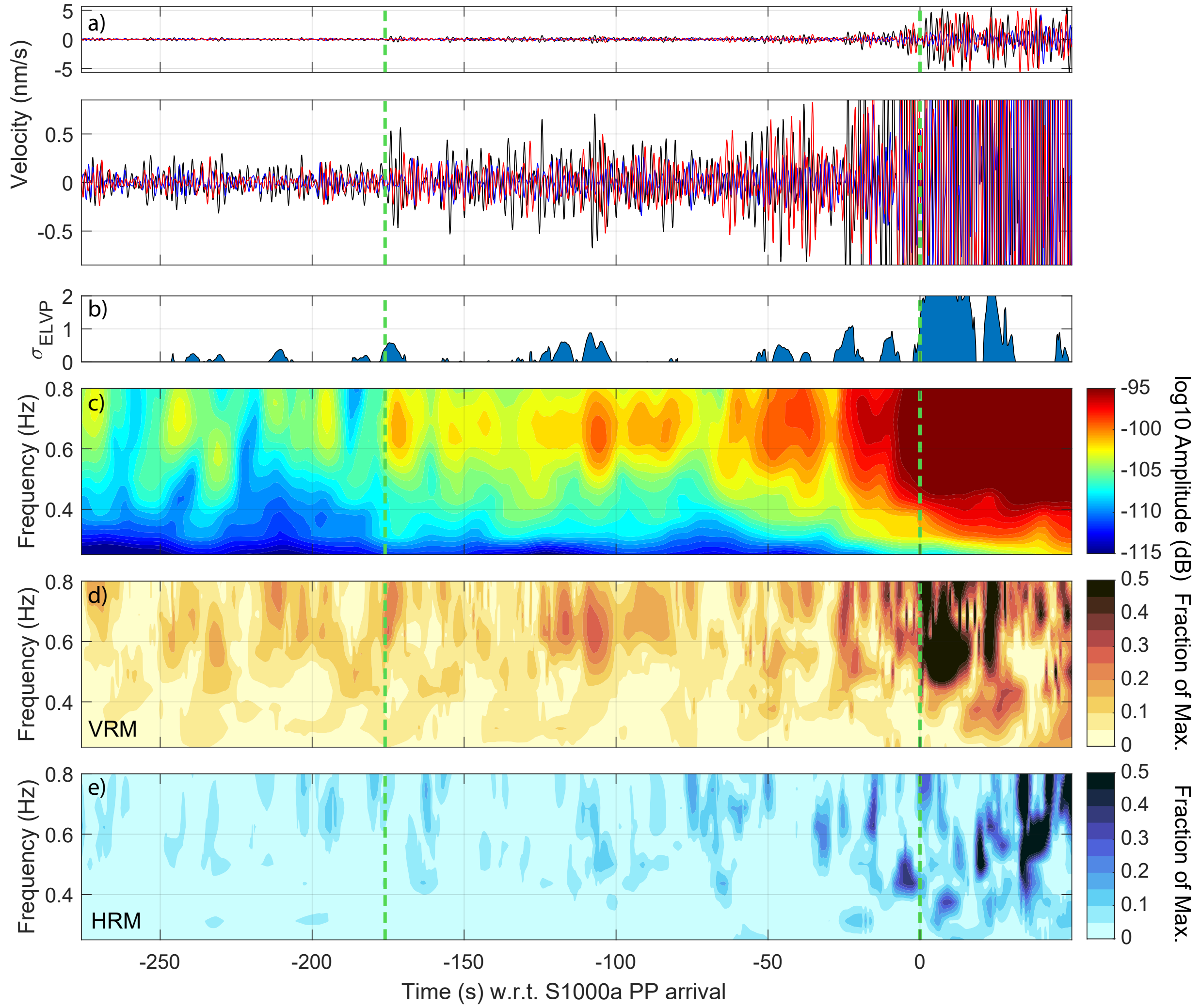

Figure 2: Frequency-dependent polarization analysis of S1000a waveforms. (a) Vertical ground velocity band pass filtered in the 0.3-0.8 Hz range, with the second panel's vertical limits revealing the smaller amplitude arrivals before the main PP arrival. (b) Z score of the excess vertically-polarized rectilinear motion, computed from panels d and e integrated across frequencies. (c) Amplitude spectrogram of vertical ground velocity. (d) Vertically-polarized and (e) horizontally-polarized rectilinear motion – defined in supplement of Stähler et al. [2021] – scaled by the square root of the spectral amplitude in each time-frequency tile. The time axis is with respect to the PP pick from Irving et al. [2023], which is, like the PbdiffPcP arrival, marked with a dashed green line.

1.2 ScS picking for event S1222a

We picked the ScS arrival of S1222a using waveform matching [*e.g.*, Stähler et al., 2021] and polarization analysis [*e.g.*, Haney et al., 2020]). In waveform matching, we utilize a 22.5 s-long time window, starting at the S arrival time from the InSight Mars Quake Service (MQS), as the template, and then in the S-coda, we search for arrivals sharing a similar shape of the template. We apply waveform matching to both the radial-

and transverse-component recordings of event S1222a, and we identify a ScS candidate at 258˜s after the S arrival (*i.e.*, from 473 s to 495.5 s after the P in Fig. 3). We then check the polarization of this candidate. The polarization analysis indicates this candidate possessing an almost horizontal particle motion (485-490 s in the fourth row in Fig. 3), which means this candidate propagates vertically to InSight, a characteristic property of ScS. Thus, we determine that this candidate is the ScS. Meanwhile, the azimuth estimate of this candidate is about 90 degrees at 0.4 Hz (480-490 s in the fourth row in Fig. 3), the energy peak frequency (the second row in Fig. 3). Since this azimuth estimate is close to the S1222a azimuth estimate (101 degrees), we adopt the radial component (*i.e.* Sv) of event S1222a S and ScS in the Q estimation.

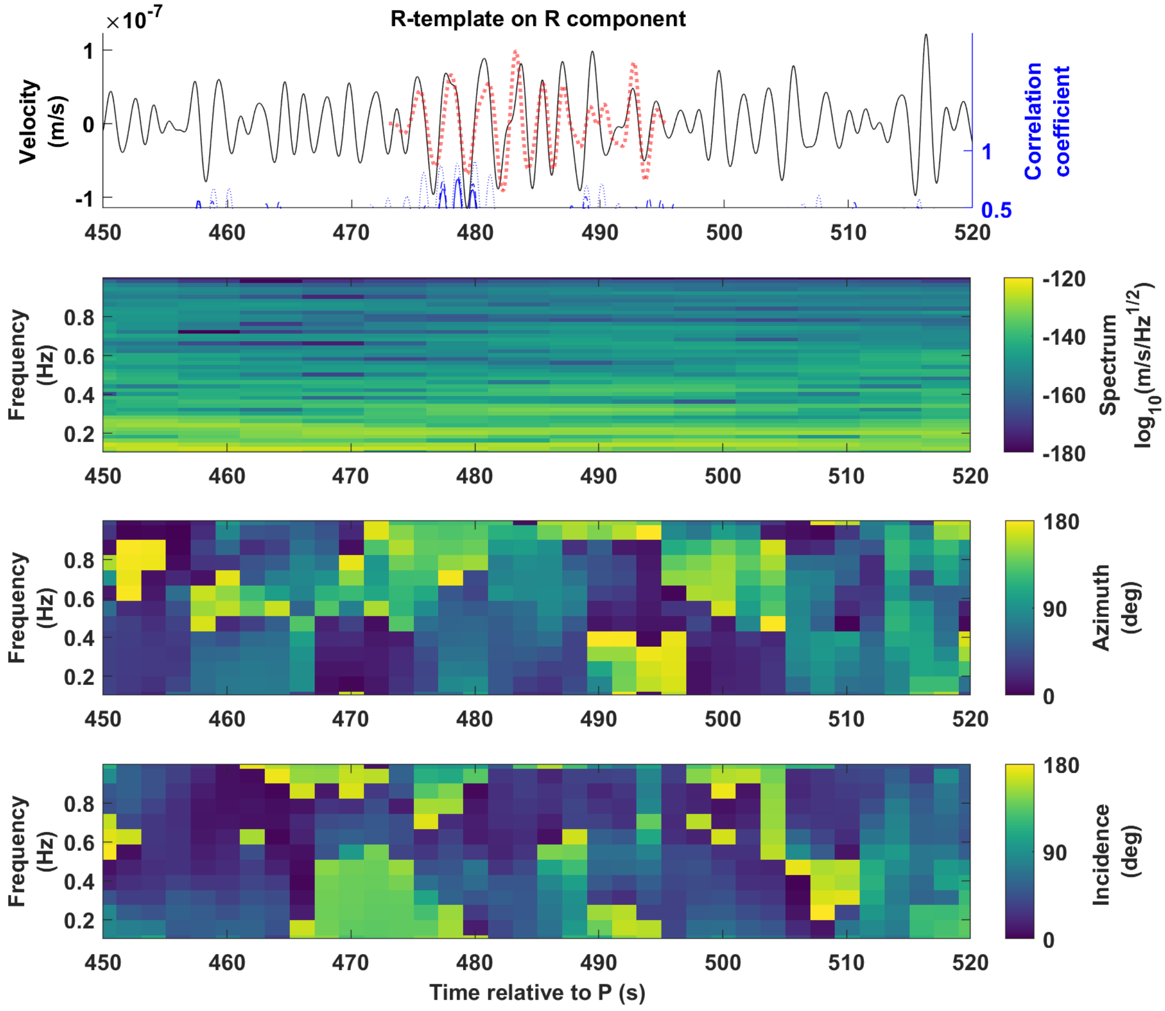

Figure 3: Waveform matching (first row) and polarization analysis (third and fourth rows) of event S1222a. In the first row, the radial component data (black) is bandpass filtered between 0.3 and 0.9 Hz, and the red dotted wiggle is the S waveform template. The second row is the spectrum of the radial component data bandpass filtered between 0.01 and 1 Hz.

2 Identification of the PbdiffPcP phase

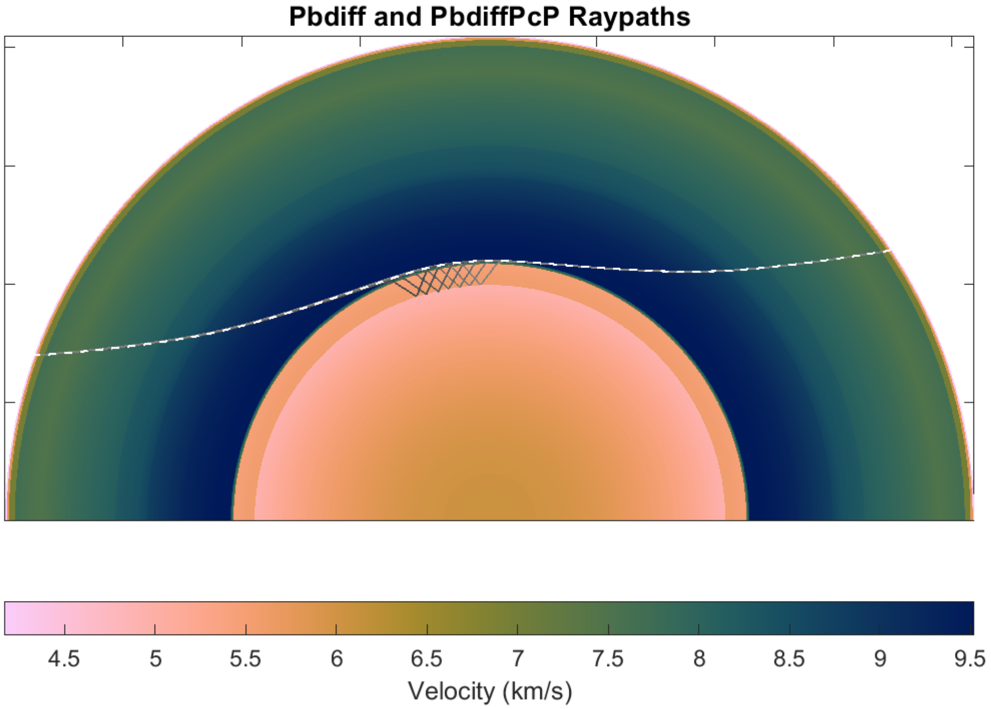

Figure 4: Raypaths through Mars for diffracted phases in a representative BML model (displayed in panels (g) to (n) of Fig. 1). Background colours indicate compressional-wave velocities throughout Mars. The white dashed line indicates the path of a wave diffracted at the base of the solid mantle (Pdiff, which on Earth diffracts along the core-mantle boundary). The grey lines highlight a selection of possible paths for PbdiffPcP, whose paths are described in detail below, and which exactly follow the Pdiff path in the mantle.

The complex velocity structure of the BML requires that we consider where compressional-wave phases are most likely to diffract as they reach the base of the mantle. We investigate physically-possible raypaths by solving the Eikonal equation using the fast marching method [Sethian and Popovici, 1999; Peyre, 2009]. To compute the raypath of the directly arriving diffracted wave, we place a source at the surface a distance of 126 degrees from the receiver (*i.e.*, at the location of S1000a), and find that the first-arriving diffracted phase for a source at an appropriate distance is Pdiff - a phase which in the BML models diffracts along the base of the completely solid (highest velocity) region at the base of the mantle (Fig. 4). We identify the diffracted phase observed in the seismic data as phase as PbdiffPcP; the 'b' indicates diffraction at the BML. We use the label 'PbdiffPcP' to refer to a compressional wave which travels through the mantle to the base of the solid layer, and then diffracts along the interface between solid and partially molten material. While diffracting, the wave radiates energy along its path; some of this energy travels down through the mushy and molten silicate layers, before being reflected back up to the diffraction path. This raypath is calculated by setting off a source at the core-mantle boundary, and follows that of Pdiff exactly. Figure 4 shows a number of such possible paths; the energy identified on the seismogram results from constructive interference of energy traveling along all such paths, which have equal travel times. Thus, some parts of the PbdiffPcP wave-packet will have taken a path which could be labelled as PbdiffPcPbdiff if they diffract both before and after travelling down to the silicate-iron interface, while others could be termed PcPbdiff if they diffract only after travelling to the silicate-iron interface. The different paths all contribute to the observed wavepacket, and we use the label PbdiffPcP to refer to them collectively.

3 Inversion approach

Our inversion method follows the approach described in [Drilleau et al., 2020, 2021] and recently applied in Stähler et al. [2021]; Drilleau et al. [2022]; Irving et al. [2023]. It relies on a geodynamic parameterization in terms of quantities that influence the thermo-chemical evolution of the planet. The latter is composed of a liquid iron core, a silicate mantle (with or without a BML) with a viscosity η follows an Arrhenius law with dependence on temperature T and pressure P (*e.g.*, Ranalli [1995]; Karato and Wu [1993]):

$$\eta = \eta_0 \exp\left(\frac{E^* + PV^*}{R\,T} - \frac{E^* + P_{\rm ref}V^*}{R\,T_{\rm ref}}\right), \quad (1)$$

where the effective activation energy E^* expresses the temperature sensitivity, the effective activation volume V^* controls the pressure dependence, R is the gas constant, and η_0 is the reference viscosity corresponding to the reference temperature $T_{\rm ref}$ = 1600 K and reference pressure $P_{\rm ref}$ = 3 GPa. The silicate envelope includes an evolving lithosphere and crust. The thermo-chemical evolution is computed following the approach described in Samuel et al. [2019] and Samuel et al. [2021] for cases with a BML. The regular silicate mantle has the composition EH45 [Sanloup et al., 1999], which yields a good match to MoI estimates and constraints on crustal thickness from receiver function analysis and gravity and topography data [Knapmeyer-Endrun et al., 2021; Wieczorek et al., 2022]. Following Irving et al. [2023], we assume that the core is well mixed, hence we model its elastic properties using a third-order isentropic Birch-Murnaghan Equation of State (EoS). With this EoS the density and acoustic velocity within the core can be computed from its density ($\rho_{\rm CMB}$), its isentropic bulk modulus (K_S), and its derivative with respect to pressure K'_S given at CMB pressure and temperature, regardless of the composition of the core.

As in Samuel et al. [2019, 2021] and reference therein, at each given mantle depth, the degree of melting (ϕ) corresponds to the ratio of super-solidus temperature divided by the temperature difference between the liquidus and the solidus. When the melt fraction exceeds a critical value, assumed to be 65% [Costa et al., 2009], the rheology is dominated by the liquid phase and therefore we assume that the shear modulus G=0 Pa, leading to $V_{\rm S}$=0 m/s. Below the critical melt fraction the mushy material has G and $V_{\rm S}$ that decrease with the melt fraction but remain > 0. In the mushy material ($\phi \leq 0.65$), we adopt a linear decrease of P and S velocities with melt fraction. In the essentially molten material ($\phi > 0.65$), $V_{\rm P}$ corresponds to the minimum bound for the bulk modulus of silicate $K_{\rm liq}$ = 24 GPa liquids with a pressure dependence $K'_{\rm liq} = 6$ from Matsui [1996] .

Fixing the values of $K_{\rm liq}$ and $K'_{\rm liq} = 6$ strongly determines V_P in the BML, which has a strong impact on the propagation seismic phases in the vicinity of the CMB, particularly for the P-diffracted phase. For example, these aforementioned quantities can determine the sign of the velocity jump across the CMB that may directly influence the raypaths of seismic phases. With the values chosen above, the seismic velocity jump going from the mantle to the core is almost systematically negative. Yet, the equation of state of silicate liquids (hence in V_P in the fully molten silicate layer) are not well constrained [Jing and ichiro Karato, 2008] and could also result in positive seismic velocity jumps across the CMB. To account for that, we considered a supplementary set of BML inversions where the bulk modulus and its pressure derivatives are have smaller values [Jing and ichiro Karato, 2008]: $K_{\rm liq}$ = 20 GPa $K'_{\rm liq} = 4.5$. This leads to smaller V_P values in the fully molten silicate part and therefore allows for cases where V_P increases when entering the core.

Along the inversion process these parameters are inferred by seismic data and by constraints on the mass of Mars Konopliv et al. [2020], which must be satisfied for each model. The present-day thermal profile of the planet is used to compute the density and velocity profiles, relying on a Gibbs-free energy minimization approach for the mantle [Connolly, 2009] (see Drilleau et al. [2021] for further details). In addition to the temperature profile, the geodynamic parameterization yields the present-day lithospheric (D_l) and crustal ($D_{\rm cr}$) thicknesses. Since the origin time of the seismic events remains unknown, we used differential seismic travel times to compute the cost function [Drilleau et al., 2022]. The moment of inertia factor and the degree-two Love number estimates were computed for each model and included in the cost function, and are compared to the estimates of Konopliv et al. [2020]. In total, 21 seismic events were considered (Table 1). For each sampled model, the body wave arrival times were computed using the `TauP` toolkit [Crotwell et al., 1999]. As in Drilleau et al. [2022] the epicentral distances and the depths of the seismic events were randomly sampled using Gaussian distributions, except for the two meteorite impacts (S1000a and S1094b) that were located precisely by orbital imaging Posiolova et al. [2022].

The parameters we invert for are those listed in Drilleau et al. [2022]. The BML inversion set requires two additional parameters to invert for: the thickness (D_d) of the BML, and its thermal conductivity, k_d. The former was randomly sampled between 100 km and 600 km while the latter was sampled between 4 and 16 W m^{-1} K^{-1} using Gaussian distributions. However, the resulting *posterior* 1-σ range for k_d was similar to the regular mantle value that we fixed to 4 W m^{-1} K^{-1}.

3.1 Note on seismic datafit and inversion results

PP and SS phases are most sensitive to the velocity structure in the vicinity of the ray turning points, which correspond to depths of $\sim$ 800 km and $\sim$ 400 km for event S1000a, respectively. The presence of a BML reduces the present-day mantle temperature, leading to faster seismic wave speeds (Fig. 3a-d). Therefore, the PP phases that are sensitive to the mantle structure have shorter travel times when a BML is present. Moreover, the lithosphere is thinner in presence of a BML (Fig. 1), leading to larger (absolute) temperature at shallow depths , and larger velocity gradients. Consequently, at shallow depths, V_S is smaller when a BML is present, implying longer SS travel times. Altogether, the presence of the BML shifts the differential travel time t_{SS}-t_{PP} towards larger values that are more compatible with the data, as seen in Fig. 3. These two mechanisms described above yield an overall good datafit when a BML is present, while this pair of differential travel times cannot be explained by a compositionally homogeneous mantle. The influences described above also explain the larger increase in mantle viscosity with pressure (larger V^* values) for BML models. Indeed, if V^* was smaller for BML models, the lithosphere thickness would be even thinner. This would result in further seismic velocity reduction at shallow depth that would increase the PP travel time (t_{PP}). Consequently, this would shift the differential travel times of $t_{\mathrm{PP}} - t_{\mathrm{Pdiff}}$, $t_{\mathrm{PP}} - t_{\mathrm{SKS}}$, and $t_{\mathrm{SS}} - t_{\mathrm{PP}}$ away from the observational ranges (Fig. 3d,f).

Despite the hotter core in BML models, the associated P-wave velocities remain similar to the non-BML models (Fig. 1e,k). This is due to the core-traversing phases Irving et al. [2023] considered in our seismic dataset, which constrain the velocity in the core. To reach similar V_P values the core for BML models is more compressible (*i.e.*, its bulk modulus is $\sim$15% smaller) than that of the non-BML cases (Table 1) in addition to be more dense (Fig. 1d,j).

4 Synthetic seismograms

Using the spectral element code AxiSEM [Nissen-Meyer et al., 2014], we computed synthetic seismograms for three models representative of the non-BML, main BML, and supplementary BML inversion sets described in Table 1 (Fig. 5a,b,c). We superimposed the predicted body arrival times on the synthetics seismograms, estimated using the TauP toolkit [Crotwell et al., 1999].

To simulate the waveform of event S1000a, we first considered an explosive seismic source located at the surface near 126° epicentral distance. The vertical component synthetics (in velocity) are displayed in Fig. 5d,e,f for epicental distances between 123° and 129°. For the three sets of models, the seismograms show the P-wave diffracted at the mantle/BML interface (Pdiff, dashed blue lines). The core bouncing diffracted P-wave (PbdiffPcP, red lines) is only observed for the two models with BML (Fig. 5e,f). Assuming a positive velocity jump at the CMB like in the supplementary BML inversion set (Fig. 5f) allows to slightly increase the amplitude of the PbdiffPcP. It is worth noting that the ray parameters of the Pdiff and the PbdiffPcP are similar on the synthetic seismograms, indicating that both seismic phases are diffracted at the mantle/BML interface.

To compute ScS waveforms displayed in panels (g), (h) and (i) of Fig. 5, we considered a marsquake located at 5 km depth generated by a normal fault, as suggested in Brinkman et al. [2021] and in Jacob et al. [2022] for events located near Cerberus Fossae. To reduce the amplitude of surface waves, the bulk and shear dissipation parameters are set to 100 in the crust. The predicted ScS arrivals are shown in magenta. Note that in the case of BML models (Fig. 5b,c), the ScS phases are reflected at the top of the liquid part of the BML, and not at the CMB, whereas they are reflected at the CMB for the non-BML model (Fig. 5a). For the three sets of BML models, P, S, and ScS arrivals are clearly visible on the radial component waveforms.

Overall, the synthetics displayed show that BML models can reproduce signals that are consistent with the arrival times picked on InSight's seismic record for deep reflected S-wave and deep and diffracted P-waves, including PbdiffPcP (see Section S 2).

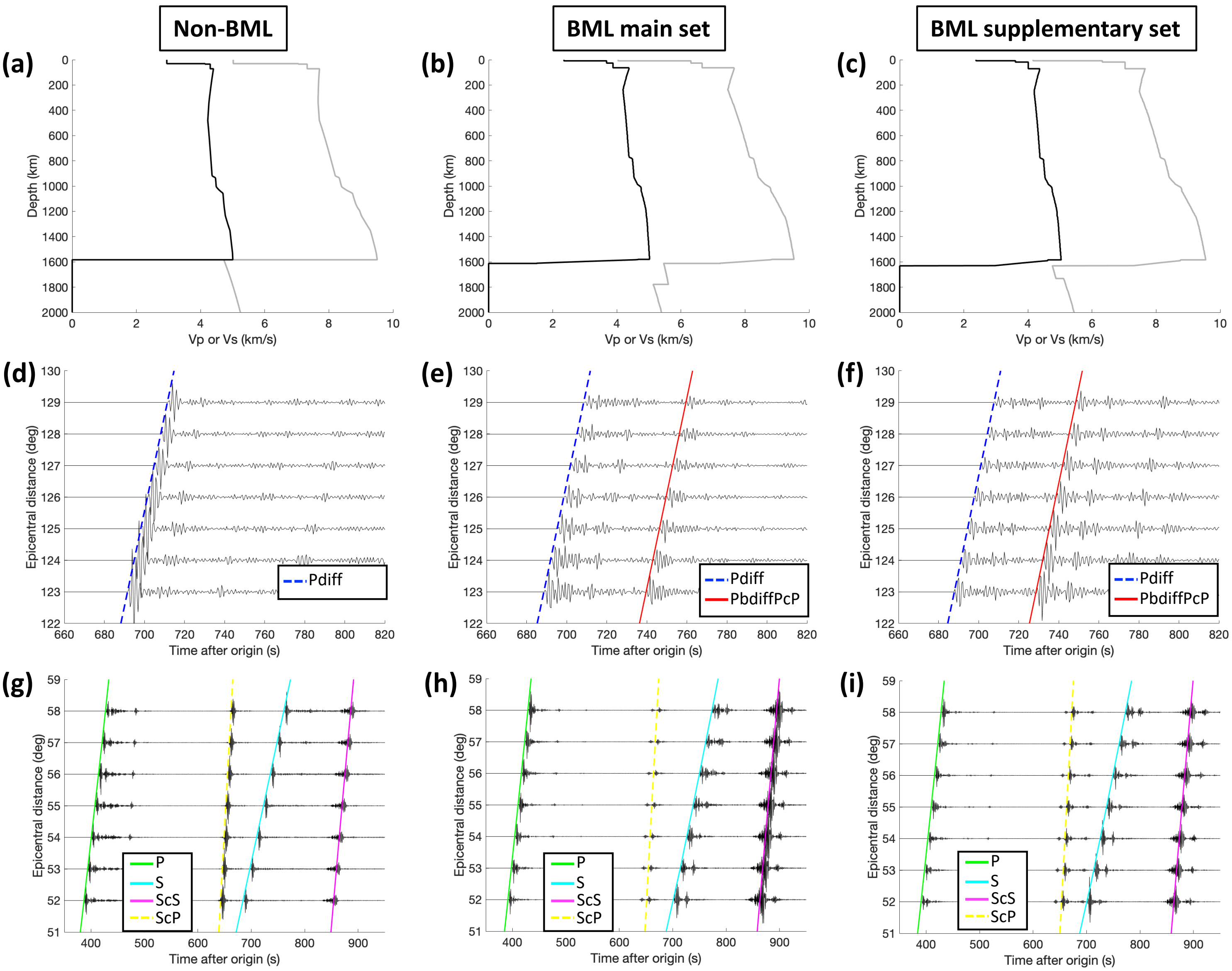

Figure 5: Examples of velocity models of the non-BML (a), main BML (b) and supplementary BML (c) inversion sets described in Table 1. (d,e,f) are synthetic seismograms showing the vertical velocity for the three sets of models between 123° and 129° of epicentral distances (close to the epicentral distance of S1000a), considering an explosion located at the surface. (g,h,i) are synthetic seismograms showing the radial velocity for the three sets of models between and epicentral distance ranging between 52° and 58°, considering a marsquake located at 5 km depth generated by a normal fault. The predicted body wave arrival times are displayed with coloured lines. The dashed lines correspond to seismic phases, which are not yet observed in the InSight recordings. The synthetic seismograms are filtered between 0.5 and 1 Hz.

5 Q estimation from direct arrivals

To estimate the apparent shear attenuation along direct S-wave raypath we selected impact S1094b (of epicentral distance 58.5 degrees) located relatively close to the source of event S1222a. We fit the observed spectra $S(\omega)$ for S1094b with a source time function and attenuation:

$$S(\omega) = \Omega_0 \left[1 + \left(\frac{\omega}{\omega_0} \right)^2 \right]^{-3/2} \exp \left(- \frac{\omega t}{2 Q_S} \right), \quad (2)$$

where Ω_0 is corresponds to the flat ('DC') value of the spectrum, Q_S is the apparent S-wave quality factor, t is the propagation time. The source time function is the *omega-3* model proposed in Garcia et al. [2022]. The corner frequency ω_0 was fixed to 3 Hz for the P-wave [Posiolova et al., 2022]. Under the assumption of a Poisson elastic medium we fixed the S-wave corner frequency to $1/\sqrt{3}$ for that of the P-wave.

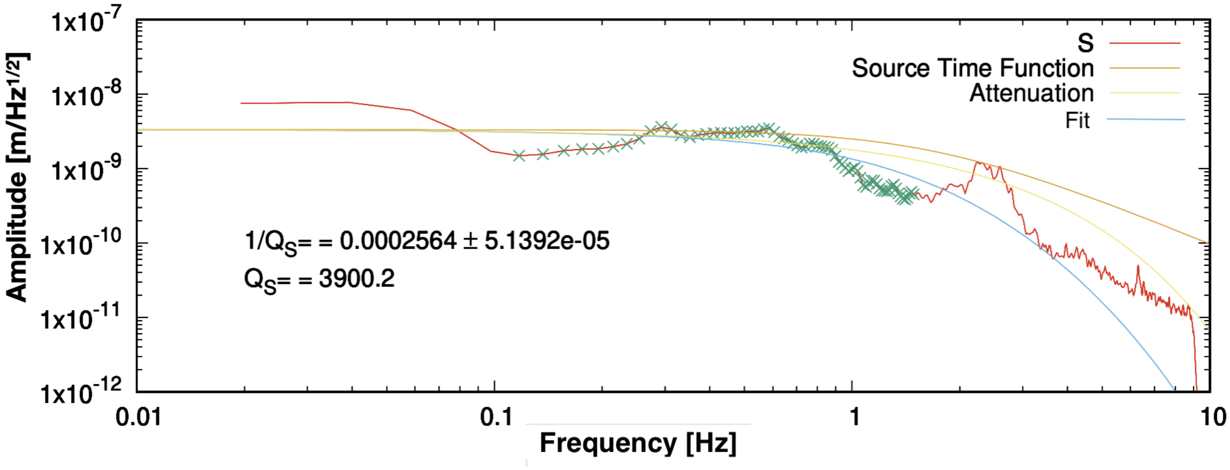

Figure 6: S-wave spectra for impact event S1094b. The red lines is observed spectra computed with 30-second time window and green crosses are the data used for the fit. The blue curve corresponds to the fit. A peak at 2.4 Hz was masked since this is likely a local effect, which is not taken into account in the model above. In addition to the final spectral shape, contribution from the source time function (orange curve) and the attenuation (yellow curve) are displayed.

The fit, displayed in Fig. 6 yields an apparent Q_S value of 3900 and a range for Q_S between 3333 and 5000.

6 Nutation of BML models

The well-known tidal forcing of the Sun and other planets, as well as the Martian Moons, induce a periodic nutation of the rotation axis of Mars. The nutation can be affected by the presence of the liquid core inside the planet since the fluid core can have a relative rotation with respect to the solid mantle. This misalignment, characterized by the Free Core Nutation (FCN) rotation normal mode, depends on the main moments of inertia of the core and on its capacity to deform due to tidal forcing and rotational rate variations. Because of the FCN the nutation can be resonantly amplified by the tidal forcing. The amplification strength is characterized by the core amplification factor F, which is related to the moment of inertia of the core, and the period of the FCN [*e.g.* Dehant and Mathews, 2015].

Recent measurements by the RISE experiment [Folkner et al., 2018] on InSight have shown that both the FCN period and F can be reliably estimated estimated by tracking the position of the InSight lander on the surface of Mars. The obtained results not only confirm previous estimates about the core structure obtained from measuring tides [Yoder et al., 2003] and reflected shear waves at the core-mantle boundary [Stähler et al., 2021], but also provide unique information about the principal moments of inertia of the core, the density jump at the core-mantle boundary, and the presence of mass anomalies related to thermal and compositional anomalies buried deep within the interior [Le Maistre et al., 2023]. In that study, it has been shown that models with a BML layer can also agree with the RISE observation. However, these models

were not constrained by the more recent seismic data used in the present study. Here we compare the FCN period and F factor of a subset of models from this present study to the estimates obtained by RISE.

To model the nutation and relative rotation of the core, two end-member scenarios are conceivable: the metallic core rotates with respect to the mantle that includes the whole BML or the metallic core rotates together with the fluid part of the BML with respect of the solid mantle and BML mushy layer. The first scenario implies that the rotating core, which has a radius smaller than 1690 km, has an F factor or core moment of inertia that is significantly lower than that determined by RISE (results not shown), and is therefore rather unlikely. For the second scenario, the models agree with both the FCN and F estimate of RISE (see Fig. 7), where the subset of models with the largest effective (R_l) core radius provide the best match. As discussed in Le Maistre et al. [2023], the depth of internal mass anomalies affect the core moment of inertia and the period of the FCN. Placing a mass anomaly at Moho depth or at the bottom of a very thick lithosphere (> 550 km) has a substantial effect on the FCN. The models used in this study have a lithosphere that is not much thicker than 300 km and placing a mass anomaly at that depth has only a small effect compared to a load at the Moho depth [Le Maistre et al., 2023]. For this reason Fig. 7 only shows the results for the latter case.

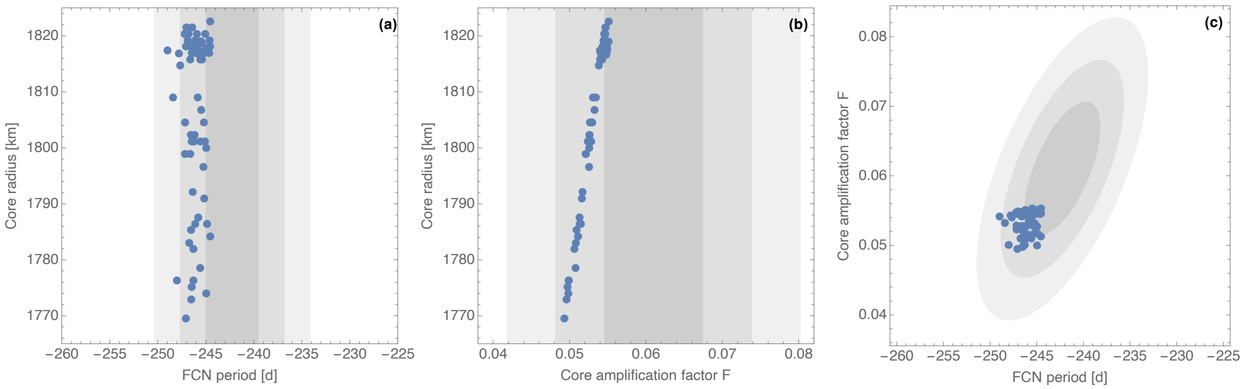

Figure 7: (a) Apparent (*i.e.,* liquid) core radius (R_l) as a function of FCN period, (b) apparent core radius as a function of core amplification factor F, and (c) FCN period-core amplification correlation. The gray shaded areas represent 1 σ, 2 σ, and 3 σ uncertainty regions.

Our results displayed in Fig. 7 show that not all models fit the RISE data equally well. This indicates that the results presented in this study could be further refined if both seismic data and RISE data were use together in a joint inversion.

7 Core formation modelling

We calculated Martian core compositions using multi-stage core formation modeling [Wade and Wood, 2005] based on the numerical code described in Badro et al. [2015]. This is the standard model of core formation on terrrestrial planets where accretionary material (could be differentiated or undifferentiated) is added successively to form Mars. At each accretional step, the added material fully melts in the primordial Martian magma ocean; and the immiscible metal and silicate separate, the metal sinks to the bottom of the magma ocean while equilibrating chemically (see Section 7.1) with the surrounding silicate, and then reaches the core, leaving behind its chemical imprint on the magma ocean, and inheriting its own composition from that chemical interaction.

7.1 Thermodynamic model of element partitioning between metal and silicate

To determine the composition of the core, we need to know how elements partition between metal and silicate during core formation. Chemical equilibrium between metal and silicate fixes the partitioning of elements between the two phases. The metal-silicate partition coefficient for an element i is:

$$D_i = \frac{X_i^{\text{metal}}}{X_i^{\text{silicate}}}, \tag{3}$$

where and X_i are molar concentrations of element i in the metal or silicate.

Carbon and sulphur are very siderophile, and since we are only interested in their abundance in the core, we simply assume that the bulk content on the planet is in the core. Nickel and cobalt are also siderophile, but we are especially interested in their mantle abundances (trace levels) because these will be used to eliminate core formation models that do not match their observed mantle abundances; in this case we will use the full thermodynamic calculation of their partitioning and abundance. We shall proceed similarly for oxygen and silicon, since their concentration in the core depends strongly on their partitioning behaviour during core formation. For the sake of our thermodynamic calculations, the elements of interest here (namely Ni, Co, Si and O) partition between core and mantle according to the following chemical reactions:

$$\begin{aligned} &\text{Ni}^{\text{metal}} + \text{FeO}^{\text{silicate}} \Longleftrightarrow \text{NiO}^{\text{silicate}} + \text{Fe}^{\text{metal}} \\ &\text{Co}^{\text{metal}} + \text{FeO}^{\text{silicate}} \Longleftrightarrow \text{CoO}^{\text{silicate}} + \text{Fe}^{\text{metal}} \\ &\text{Si}^{\text{metal}} + 2\text{FeO}^{\text{silicate}} \Longleftrightarrow \text{SiO}_2{}^{\text{silicate}} + 2\text{Fe}^{\text{metal}} \\ &\text{O}^{\text{metal}} + \text{Fe}^{\text{metal}} \Longleftrightarrow \text{FeO}^{\text{silicate}} \end{aligned} \tag{4}$$

At thermodynamic equilibrium, the partition coefficient associated with these reactions can be parameterised (for the full thermodynamic formalism, see [Gendre et al., 2022]) as:

$$\log(D_i) = a + \frac{b}{T} + c\frac{P}{T} - \frac{n}{2}\log\frac{X_{\text{FeO}}^{\text{silicate}}}{X_{\text{Fe}}^{\text{metal}}} - \log\gamma_i + \frac{n}{2}\log\gamma_{\text{Fe}}, \tag{5}$$

where i is the element of interest (Ni, Co, Si, O), a, b, and c are the entropy, enthalpy, and volume changes due to the partition of element i between metal and silicate (reported in Table 3). $X_{\text{FeO}}^{\text{silicate}}$ and $X_{\text{Fe}}^{\text{metal}}$ are the FeO concentration in the silicate and Fe concentration in the metal. n is the valence of element i in the silicate melt; γ_{Fe} and γ_i are the activity coefficients of iron and element i in the metal.

The activity coefficients are calculated via an approach that relies on interaction parameters, ε, which are used to calculate the activity of multicomponent metallic solutions. In a metallic solution consisting of N components, the activity coefficients of Fe and the remaining N-1 solutes (i) are given by:

$$\begin{aligned} \ln\gamma_{\text{Fe}} = &\sum_{i=1}^{N-1}\varepsilon_i^i(X_i + \ln(1-X_i)) \\ &- \sum_{j=1}^{N-2}\sum_{k=j+1}^{N-1}\varepsilon_j^k X_j X_k\left(1 + \frac{\ln(1-X_j)}{X_j} + \frac{\ln(1-X_k)}{X_k}\right) \\ &+ \sum_{i=1}^{N-1}\sum_{\substack{k=1\\k\neq i}}^{N-1}\varepsilon_i^k X_i X_k\left(1 + \frac{\ln(1-X_k)}{X_k} - \frac{1}{1-X_i}\right) \\ &+ \frac{1}{2}\sum_{j=1}^{N-2}\sum_{k=j+1}^{N-1}\varepsilon_j^k X_j^2 X_k^2\left(\frac{1}{1-X_j} + \frac{1}{1-X_k} - 1\right) \\ &- \sum_{i=1}^{N-1}\sum_{\substack{k=1\\k\neq i}}^{N-1}\varepsilon_i^k X_i^2 X_k^2\left(1 + \frac{1}{1-X_i} + \frac{1}{1-X_k} + \frac{X_i}{2(1-X_i)^2} - 1\right) \end{aligned} \tag{6}$$

and,

$$
\begin{aligned}
\ln \gamma_i = &\ln \gamma_{\mathrm{Fe}} + \ln \gamma_i^0 \\
&- \varepsilon_i^i \ln(1 - X_i) \\
&+ \sum_{j=1(j\neq i)}^{N-1} \varepsilon_i^j X_j \left(1 + \frac{\ln(1-X_j)}{X_j} - \frac{1}{(1-X_i)}\right) \\
&+ \sum_{j=1(j\neq i)}^{N-1} \varepsilon_i^j X_j^2 X_i \left(\frac{1}{(1-X_i)} + \frac{1}{(1-X_j)} + \frac{X_i}{2(1-X_i)^2} - 1\right).
\end{aligned}
\tag{7}
$$

Values of the interaction parameters $\varepsilon(T_0)$ are reported at a reference temperature of T_0 =1873 K (Table 2), and scale with T as:

$$
\varepsilon(T) = \varepsilon(T_0)\frac{T_0}{T}. \tag{8}
$$

ε	O	Si	S	Ni	Co
O	-4.5	-5.0	-6.1	1.4	1.9
Si	-5.0	12.4	9.0	1.2	0.0
S	-6.1	9.0	-5.7	0.0	0.6
Ni	1.4	1.2	0.0	0.1	0.0
Co	1.9	0.0	0.6	0.0	1.2

Table 2: Interaction parameters from [Badro et al., 2015; Gendre et al., 2022] used in the thermodynamic modelling of metal-silicate partitioning, reported at a reference temperature of 1873 K.

Element	a	b (K)	c (K/GPa)	n	$\ln \gamma_i^0(T)$
Ni	0.304 (0.162)	2916 (344)	-60 (5)	+2	$-0.42 \cdot 1873/T$
Co	0.287 (0.141)	1360 (286)	-35 (5)	+2	$-0.60 \cdot 1873/T$
Si	0.364 (0.28)	-16520 (716)	0	+4	$-6.65 \cdot 1873/T$
O	2.736 (0.14)	11439 (387)	0	-2	$4.29–16500/T$

Table 3: Thermodynamic parameters of Equation 5 for Ni ,Co, O, and Si from [Badro et al., 2015; Gendre et al., 2022], values in parenthesis are 1-sigma uncertainties.

7.2 Melting of bulk silicate Mars

The solidus of bulk silicate Mars are taken from [Duncan et al., 2018], and the liquidus is from [Ruedas and Breuer, 2017] (see Fig. 1c). Both equations are given below, where T is temperature in Kelvin and P is pressure in GPa.

$$
\begin{cases}
T_{\text{solidus}} = 1361 + 120.2P - 4.877P^2 & \text{if } P < 10 \text{ GPa}, \\
T_{\text{solidus}} = 2075 + 38.18(P-10) - 1.323(P-10)^2 & \text{if } P > 10 \text{ GPa}, \\
\\
T_{\text{liquidus}} = 2160.6 + 64.7109P - 3.97463P^2 + 0.0957894P^3 \ .
\end{cases}
\tag{9}
$$

7.3 Core composition

The evolutionary process of multi-stage core formation was implemented here and discretised in 100 accretional steps. At each step i, the pressure P_i at the bottom of the magma ocean is calculated as a function of the fraction of Mars' accreted mass M_i/M_{final}, and final magma ocean depth P_{final} [Badro et al., 2015; Piet et al., 2017] according to:

$$P_i = P_{\mathrm{final}} \left(\frac{M_i}{M_{\mathrm{final}}} \right)^{2/3}, \tag{10}$$

where P_{final} is a free parameter, but obviously comprised between 0 (the surface) and 19 GPa, the pressure at the core-mantle boundary (maximum depth, global magma ocean).
The temperature in the magma ocean is calculated using the melting curves (solidus, liquidus) [Duncan et al., 2018; Ruedas and Breuer, 2017] for a bulk silicate Mars composition as a function of pressure (see details in 7.2) all the way down to the CMB. The choice of this temperature profile (solidus *vs.* liquidus) is another free parameter.
The composition of bulk silicate Mars is fixed to values in [Samuel et al., 2022] of 41 mol.% SiO2 and 15.3 mol.% FeO, respectively. This value incorporates the BML and the Martian mantle. Finally, the sulphur, carbon, and hydrogen content of Mars are the last free parameters of the model.

We carried out 1200 simulations where we systematically varied the free parameters along the full extent of plausibility:

- the depth of Mars' magma ocean was varied from the surface to the core-mantle boundary;
- the geotherm in the Martian magma ocean was varied from the solidus to the liquidus of bulk silicate Mars;
- the sulphur content in the Martian core varied from 0 to 50 mol% (FeS);
- the carbon content was varied from 0 to the saturation limit in an Fe-S alloy [Tsuno et al., 2018]. Thus, while being a free parameter, the maximum carbon concentration is directly linked to that of S according to:

$$\ln \mathrm{C}^{\mathrm{core}}_{\mathrm{max}} = -33.59 - \frac{2726}{T} - 38.7\frac{P}{T} + 7.51 \ln(1 - \mathrm{S}^{\mathrm{core}}), \tag{11}$$

  where P and T are pressure and temperature, $\mathrm{S}^{\mathrm{core}}$ is the sulphur concentration in the core (in wt.%), and $\mathrm{C}^{\mathrm{core}}_{\mathrm{max}}$ the resulting maximum possible amount of carbon (in wt.%) in the core under those conditions (Fig. 7.3).
- The hydrogen content in the core is unconstrained, and can vary from 0 to an absolute upper bound of 0.15 wt% based on a recent experimental metal-silicate hydrogen partitioning study [Tagawa et al., 2022a], which we scaled from terrestrial to Martian conditions (Mars has higher FeO content and lower H_2O content in its mantle, and much lower pressure than on Earth, and all of these contribute to reducing the H content in the core). That bound is even lower, with basically no hydrogen in Mars' core, on the basis of magma ocean–primordial atmosphere equilibrium models [Young et al., 2023].

At each step of accretion and core formation, the output of the model is the composition of the metal and the silicate magma ocean resulting from this differentiation process at a given P and T. All uncertainties on the thermodynamic variables were propagated using Monte Carlo sampling (10,000 samples per point). The process is repeated until Mars is fully accreted, and the mantle and core have reached their final composition for the aforementioned tracked elements.

This approach produces 1200 models of core composition, which now need to be trimmed down. Because each model is composed of a core and a mantle, we retain only the models that produce mantle compositions consistent with Martian mantle geochemistry. Therefore, we kept only the models where Ni and Co abundances fall within the observable range; the Ni and Co depletion in the Martian mantle can be expressed conveniently by a bulk core-mantle partition coefficient of D_{Ni}=180–300 and D_{Co}=21–57 [Yoshizaki and McDonough, 2020]. We call this subset of the models the "geochemically consistent set".

Interestingly, and as previously observed [Gendre et al., 2022; Brennan et al., 2020], all core models are essentially devoid of silicon (< 0.03 wt%). We plotted the remaining core compositional space (O,C,S) in Fig. 7.3. Possible O and C concentrations are plotted *vs.* S concentration (free parameter), and show an increase of O and a decrease of C concentration with increasing S.

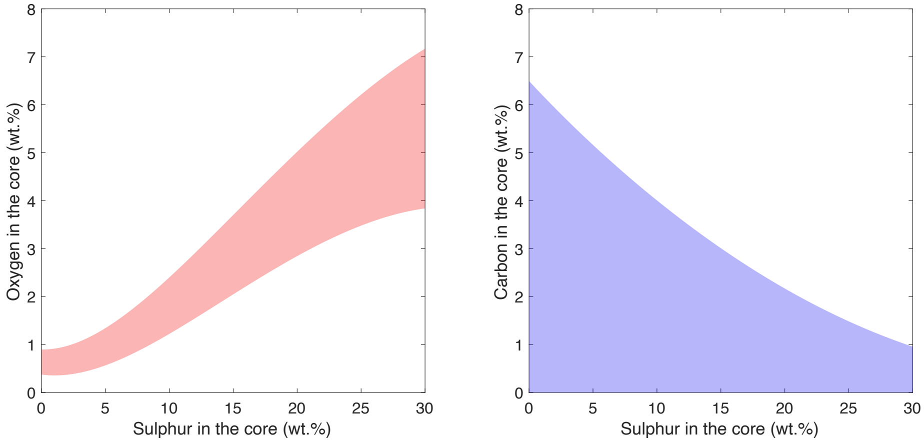

Figure 8: Composition of Mars' core from core formation modelling. (left) Oxygen, (right) carbon concentrations are plotted as a function of sulphur content, which then remains the only free compositional parameter of the problem. Silicon in always below 0.1 wt% and negligible, and hydrogen is unconstrained, but is significantly below 0.15 wt %. The Oxygen composition range is bound by the two following polynomials: $O_{\min} = -3.11676103\ 10^{-4}S^3 + 1.98176935\ 10^{-2}S^2 - 7.18932170\ 10^{-2}S + 1.28917783$ and $O_{\max} = -3.(90147450\ 10^{-4}S^3 + 2.58013870\ 10^{-2}S^2 - 2.27374258\ 10^{-2}S + 3.06338724$, and the Carbon composition range is bound by the saturation value (which depends on S and O concentration, Eq. 11) as an upper bound and 0 as a lower bound.

In terrestrial core formation modelling [Wade and Wood, 2005; Badro et al., 2015], FeO concentration in the magma ocean is another important free parameter. Here, we fixed that value to that in bulk silicate Mars of 15.9 mol%, and assumed it to be constant during accretion, which is justified by the fact that Mars formed very early during the first two million years of the Solar System [Dauphas and Pourmand, 2011]. In addition, with the relatively low pressures involved here (pressure in Mars' magma ocean is significantly lower than on Earth), a variation of FeO during accretion has negligible impact on final core composition in terms of O, S and C contents. However, it could have a larger impact of silicon, that is significantly more siderophile under more reducing conditions. Therefore, we tested a scenario where FeO concentration in the Martian mantle starts at 0 (extremely reducing) and increases as accretion proceeds to reach the final value of 15.9 mol%, akin to what was proposed for the Earth in [Wade and Wood, 2005]. In this case, the maximum silicon concentration in the core reaches a maximum value of 0.09 wt%. While this is three times the amount of the constant FeO model we have used, it still has no practical implication on the major element composition of Mars' core.

Our final range of core compositions obtained here (Fig. 7.3) is geochemically consistent, but its span can be further reduced by discriminating on the basis of the geophysical properties of the Martian core. For this, we need to model the seismologically relevant (density and bulk modulus) properties of iron alloys based on existing experimental data, apply these properties to all core compositions shown in Fig. 7.3, and only keep the ones that match the properties of the Martian core. This is developed in the next section.

8 Seismologically constrained core composition

To infer core composition from seismic data, we rely exclusively on an EoS for the liquid core built with experimental data. This is currently the only reliable approach because even the most recent alternative *ab*

initio methods fail to reproduce experimental data at conditions prevailing for Mars' core and/or rely on *ad hoc* corrections that are not physical. We describe below our EoS for the liquid core.

8.1 Equation of state for the liquid core

We model core properties (density and sound velocity) under the assumption that it is an iron alloy containing sulfur (S), oxygen (O), carbon (C), and hydrogen (H) as light elements (given in decreasing order of occurrence). All four light elements are soluble in iron at core forming conditions ([e.g. Steenstra and van Westrenen, 2018]) and abundant enough to significantly affect the density and sound velocity of the core.

We describe the EoS of the liquid Fe-S-O-C-H following Irving et al. [2023] (which we recall here for completeness). It assumes an asymmetric Margules mixing model that uses Fe, FeS, FeO, Fe_3C, and FeH as end-members. The volume of the solution writes:

$$V(\{\chi_i\}, P, T) = V_{\text{ideal}}(\{\chi_i\}, P, T) + V_{\text{ex}}(\{\chi_i\}, p)\ , \tag{12}$$

where

$$V_{\text{ideal}}(\{\chi_i\}, P, T) = \sum_{i=\{\text{Fe,FeS,FeO,Fe}_3\text{C,FeH}\}} \chi_i V_i(P, T)\ ,$$

is the ideal contribution to the volume, and

$$\begin{aligned} V_{\text{ex}}(\{\chi_i\}, P) =& \chi_{\text{Fe}}\chi_{\text{FeO}}\left(\chi_{\text{FeO}}W_{\text{Fe}-\text{FeO}} + \chi_{\text{Fe}}W_{\text{FeO}-\text{Fe}}\right) + \\ & \chi_{\text{Fe}}\chi_{\text{FeS}}\left(\chi_{\text{FeS}}W_{\text{Fe}-\text{FeS}}(P) + \chi_{\text{Fe}}W_{\text{FeS}-\text{Fe}}(P)\right) \end{aligned}$$

is the excessive contribution. In the above expressions χ_i and V_i are the molar fractions and molar volumes of Fe, FeS, FeO, Fe_3C, and FeH in their liquid state, and the W_{i_j} are the Margules coefficients for $\text{Fe} - \text{FeO}$ [Komabayashi, 2014] and $\text{Fe} - \text{FeS}$ [Xu et al., 2021].

The molar volumes of the end-members were computed using their EoS. We use Dorogokupets et al. [2017] for liquid Fe, Morard et al. [2022], liquid FeO and Tagawa et al. [2022b] for liquid FeH. The EoS for liquid FeS and Margules coefficients for $\text{Fe} - \text{FeS}$ were deduced from the elastic data used in [Xu et al., 2021] and high pressure density (up to 43 GPa) [Morard et al., 2013] and acoustic velocity data (up to 50 GPa) [Kawaguchi et al., 2017; Nishida et al., 2020]. Finally, the EoS for liquid Fe_3C was based on density and acoustic data [Terasaki et al., 2010; Morard et al., 2017; Shimoyama et al., 2016] acquired at pressures up to 53 GPa. The EoS parameters and Margules parameters for FeS and Fe_3C are given in Table 4. From the EoS of the end-members and Eq. 12 all relevant thermodynamic quantities required to compute the adiabatic gradient and the acoustic velocity in the core are obtained by applying classical thermodynamic relations.

Parameter	FeS	Fe_3C
T_0 [K]	1650	1723
V_0 [cm^3/mol]	24.4±0.3	26.5±0.1
C_p [J/K/mol]	62.5	215.
α [10^5/K]	11.8	14.8±5
K_T [GPa]	12.0±-0.8	57.5±13
K'_T	6.9±0.3	15.±3
γ	0.62±0.04	1.34±0.4
δ_T	0.4±-0.5	9.1±4.0
W_{Fe-X} [cm^3/mol]	-9.9±-1.4	-
W_{X-Fe} [cm^3/mol]	-3.54±-0.4	-
B_0 [GPa]	3.02±-0.3	-
B'_0	2.6±-0.4	-

Table 4: EoS parameters for the Anderson-Grüneisen equation (*e.g.*, [Komabayashi, 2014] and Margules coefficients. κ=1.4 and X stands for FeS or Fe_3C.

The addition of light elements to Fe decreases its density (see Fig. 8.1), with different magnitudes for each light element, as seen for Earth's core [Badro et al., 2014; Huang et al., 2019; Umemoto and Hirose, 2020].

While the relative effect is almost linear for density, its is far more complex for acoustic velocity. In particular, the pressure-dependent non-ideal mixing of liquid FeS into liquid Fe results in an initial increasing velocity that reaches a maximum and can later decreases below the velocity of liquid Fe (depending on pressure) for large fractions of S. Conversely, adding O results in an initially decreasing velocity that reaches a minimum at about 4 wt% and a subsequent increase with the amount of O above the velocity of liquid Fe. We note however that at pressure and temperature conditions of the Martian core, the relative variation with respect to pure Fe resulting from the addition of S or O are small compared to the large effect resulting from the addition of H or C.

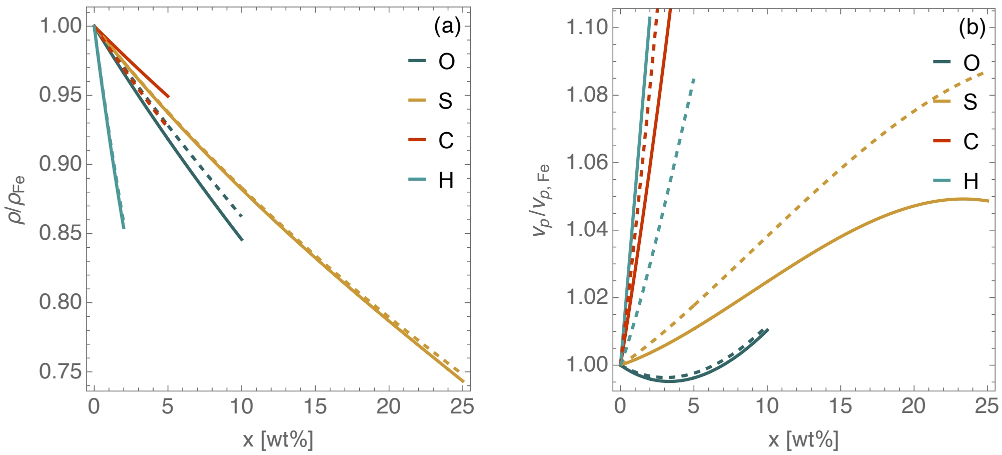

Figure 9: Effect of light element concentration x on density (a) and acoustic velocity (b) compared to iron at Mars' CMB conditions (full lines) ($P = 22$ GPa, $T = 2760$ K) and core center conditions (dashed lines) ($P = 40$ GPa, $T = 3100$ K)

8.2 Seismologically constrained core composition

Here we describe how we deduce the core compositions that best match the seismologically-inferred EoS parameters (ρ_0, K_S, and K'_S). Together with the core equation of state (8.1), they are representative for the density and acoustic velocity profiles in the whole core.

We considered three different plausible core alloys: Fe-O-S, Fe-O-S-C, and Fe-O-S-C-H. For each of these, the amount of O is related to S, and the fraction of C is below its solubility limit in Fe-S (see Sec 7.3). The remaining free parameters are the amounts of S and H. Our upper limit for S is 25 wt%, above the permissible upper geochemical limit [e.g Steenstra et al., 2018] and the amount of H is assumed below 0.5 wt% (see Sec 7.3). To deduce the amount of S and H in the liquid alloy we follow a two-step procedure: First we seek the amount of S and H such that the density of the liquid alloy at (P_{CMB}, T_{CMB}) matches ρ_0. A large fraction of the seismologically deduced models that match the density of the alloy are not compatible with the elastic properties of the alloy (see Fig. 10). For this reason, in a second step, we only retain the subset of seismic models where K_S and K'_S match those of the alloy considered here within 1%, and 15% respectively, and use this subset of models to inform about the composition of the core.

The core composition inferred from the output models for the three alloys considered are displayed in Fig. 11. A subset of our models (less than one percent of the best 1000 models) exhibit core elastic properties that can be reconciled with an Fe-O-S alloy with an average fraction of S close to the geochemically acceptable upper limit (about 17 wt%, *e.g.*, Steenstra and van Westrenen [2018]) (Fig. 11a). This contrasts with Stähler et al. [2021]; Irving et al. [2023] where the smaller seismically-derived core densities (0.5 g/cm^3 on average) requires a fraction of S well above the geochemical upper limit. The addition of C increases the population of compatible models to about 5%, and decreases the amount of S by about 3.5 wt% (Fig. 11a). Including up to 0.5 wt% of H (we allowed an upper bound for H beyond the maximum limit of 0.15 wt% (7.3) for a better understanding of the influence of this element on the fractions of other light elements) in Fe-S-O-C reduces the required amount of S by a further 1.5 wt%, with a comparable population of compatible models.

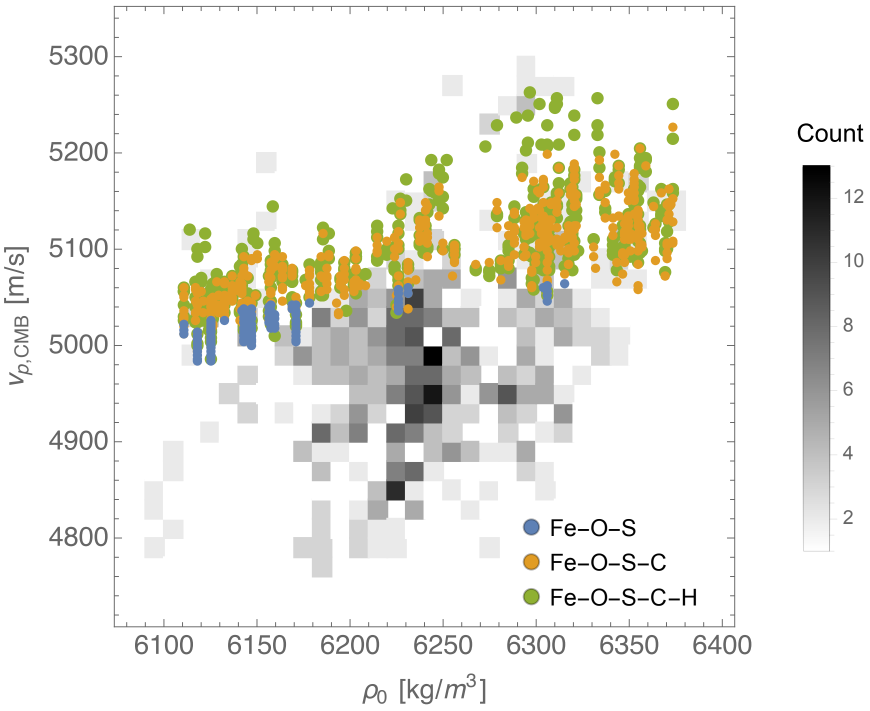

Figure 10: Core density-velocity histogram for the Mars models of this study. The blue, yellow, and green dots represent interior models that have elastic properties compatible with and Fe-O-S, Fe-O-S-H, and Fe-O-S-C-H alloy, respectively.

The average amount of H of about 0.25 wt% (Fig. 11d) is larger than the 0.15 wt% that is thought to be the upper bound for H in the core of Mars (see Section 7.3). About a quarter of the Fe-O-S-C-H models have a H fraction below the upper bound. Even though these models require on average more S (about 1 wt%), their amount of S of about 14 wt% lies within the geochemically permissible range. Note that for both Fe-O-S-C and Fe-O-S-C-H, the amount of C in the alloy (Fig. 11c) is appreciably below its solubility limit.

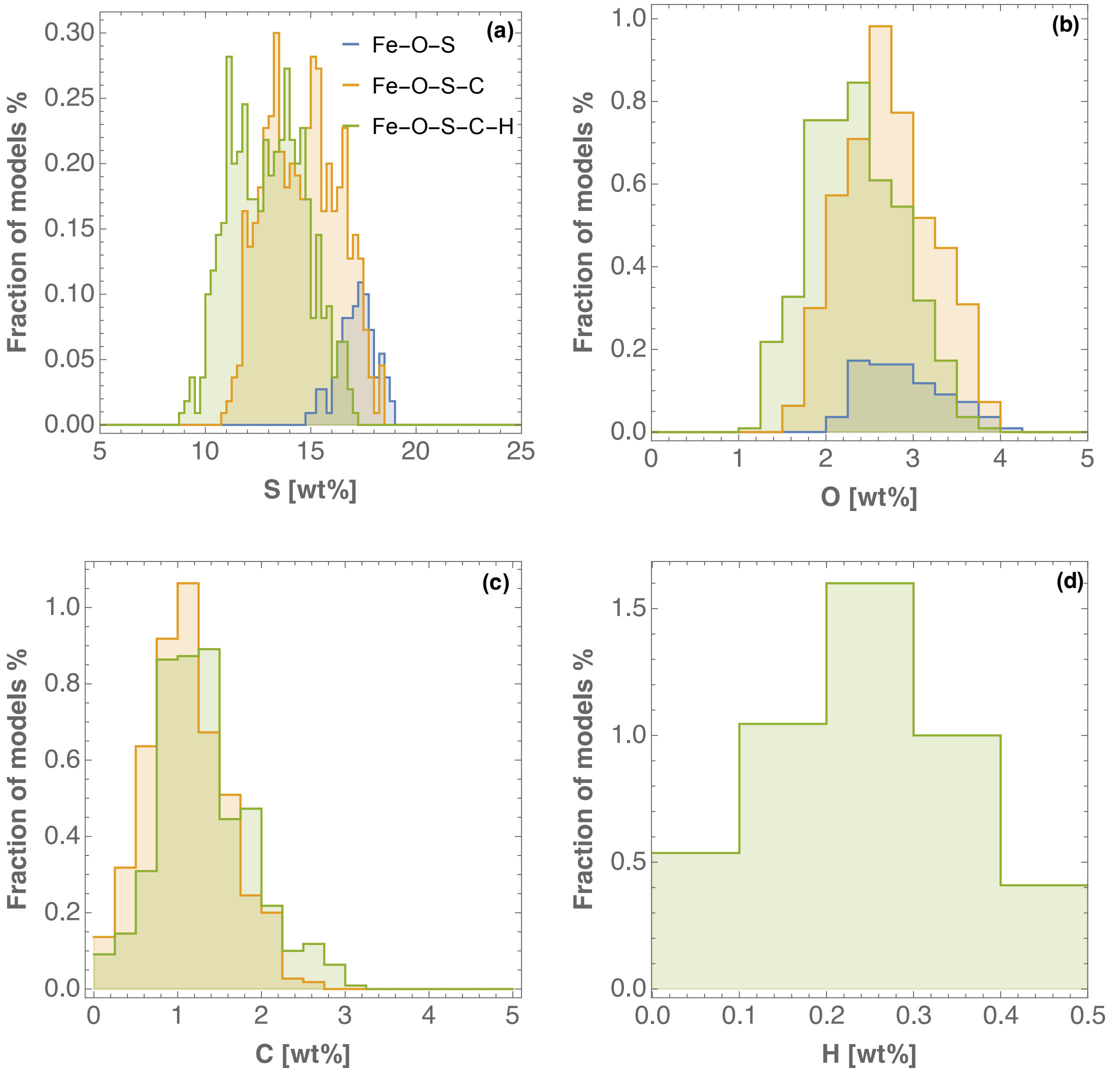

Figure 11: Core composition inferred from the output models of the BML set. Core sulfur, oxygen, carbon, and hydrogen weight fraction assuming a liquid core alloy of Fe-O-S, Fe-O-S-H, and Fe-O-S-C-H.

A small fraction of seismically inferred models from this study have elastic properties that are well reproduced by a Fe-O-S alloy with S below the acceptable upper limit of 17 wt%. This contrasts with Mars models inferred from seismic data assuming a homogeneous mantle composition Irving et al. [2023] whose core elastic properties can only be explained with an alloy that contains additionally to O and S up to 1 wt% of H if S is limited to be below 17 wt%. Larger fractions of seismically inferred models correspond to Fe-O-S-C and Fe-O-S-H assemblages (in similar proportions).

Bibliography

Badro, J., Brodholt, J. P., Piet, H., Siebert, J., and Ryerson, F. J. (2015). Core formation and core composition from coupled geochemical and geophysical constraints. *Proc. Nat. Acad. Sci.*, 112(40):12310–12314.

Badro, J., Côté, A. S., and Brodholt, J. P. (2014). A seismologically consistent compositional model of Earth's core. *Proc. Nat. Acad. Sci.*, 111(21):7542–7545.

Brennan, M. C., Fischer, R. A., and Irving, J. C. E. (2020). Core formation and geophysical properties of Mars. *Earth and Planetary Science Letters*, 530:115923.

Brinkman, N., Stähler, S. C., Giardini, D., Schmelzbach, C., Khan, A., Jacob, A., Fuji, N., Perrin, C., Lognonné, P., Beucler, E., Böse, M., Ceylan, S., Charalambous, C., Clinton, J. F., van Driel, M., Euchner, F., Horleston, A., Kawamura, T., Knapmeyer-Endrun, B., Mainsant, G., Panning, M. P., Pike, W. T., Scholz, J., Robertsson, J. O. A., and Banerdt, W. B. (2021). First Focal Mechanisms of Marsquakes. *Journal of Geophysical Research: Planets*, 126(4).

Connolly, J. A. D. (2009). The geodynamic equation of state: What and how. *g-cubed*, 10. Q10014, doi:10.1029/2009GC002540.

Costa, A., Caricchi, L., and Bagdassarov, N. (2009). A model for the rheology of particle-bearing suspensions and partially molten rocks. *g-cubed*, 10(3):1–13.

Crotwell, H. P., Owens, T. J., and Ritsema, J. (1999). The TauP Toolkit: Flexible Seismic Travel-time and Ray-path Utilities. *Seismological Research Letters*, 70(2):154–160.

Dauphas, N. and Pourmand, A. (2011). Hf-W-Th evidence for rapid growth of Mars and its status as a planetary embryo. *Nature*, 473(7348):489–492.

Dehant, V. and Mathews, P. M. (2015). *Precession, Nutation and Wobble of the Earth*. Cambridge University Press.

Dorogokupets, P. I., Dymshits, A. M., Litasov, K. D., and Sokolova, T. S. (2017). Thermodynamics and Equations of State of Iron to 350 GPa and 6000 K. *Scientific Reports*, 7:41863 EP –.

Drilleau, M., Beucler, E., Lognonné, P., Panning, M., Knapmeyer-Endrun, B., Banerdt, B. W., Beghein, C., Ceylan, S., van Driel, M., Joshi, R., Kawamura, T., Khan, A., Menina, S., Rivoldini, A., Samuel, H., Stähler, S., Xu, H., Bonnin, M., Clinton, J., Giardini, D., Kenda, B., Lekic, V., Mocquet, A., Murdoch, N., Schimmel, M., Smrekar, S., Sutzmann, E., Tauzin, B., and Tharimena, S. (2020). MSS/1: Single-station and single-event marsquake inversion. *Earth and Space Sciences*, page e2020EA001118.

Drilleau, M., Samuel, H., Garcia, R. F., Rivoldini, A., Perrin, C., Michaut, C., Wieczorek, M., Tauzin, B., Connolly, J. A. D., Meyer, P., Lognonné, P., and Banerdt, W. B. (2022). Marsquake locations and 1-d seismic models for mars from insight data. *Journal of Geophysical Research: Planets*, 127(9):e2021JE007067. e2021JE007067 2021JE007067.

Drilleau, M., Samuel, H., Rivoldini, A., Panning, M., and Lognonné, P. (2021). Bayesian inversion of the Martian structure using geodynamic constraints. *Geophysical Journal International*.

Duncan, M. S., Schmerr, N. C., Bertka, C. M., and Fei, Y. (2018). Extending the Solidus for a Model Iron-Rich Martian Mantle Composition to 25 GPa. *Geophysical Research Letters*, 45(19):10,211–10,220.

Duran, C., Khan, A., Ceylan, S., Charalambous, C., Kim, D., Drilleau, M., Samuel, H., and Giardini, D. (2022). Observation of a core-diffracted P-wave from a farside impact with implications for the lower-mantle structure of Mars. *Geophysical Research Letters*.

Folkner, W. M., Dehant, V., Le Maistre, S., Yseboodt, M., Rivoldini, A., Van Hoolst, T., Asmar, S. W., and Golombek, M. P. (2018). The Rotation and Interior Structure Experiment on the InSight Mission to Mars. *Space Science Reviews*, 214(5):100.

Garcia, R. F., Daubar, I. J., Beucler, E., Posiolova, L. V., Collins, G. S., Lognonné, P., Rolland, L., Xu, Z., Wójcicka, N., Spiga, A., Fernando, B., Speth, G., Martire, L., Rajšić, A., Miljković, K., Sansom, E. K., Charalambous, C., Ceylan, S., Menina, S., Margerin, L., Lapeyre, R., Neidhart, T., Teanby, N. A., Schmerr, N. C., Bonnin, M., Froment, M., Clinton, J. F., Karatekin, O., Stähler, S. C., Dahmen, N. L., Durán, C., Horleston, A., Kawamura, T., Plasman, M., Zenhäusern, G., Giardini, D., Panning, M., Malin, M., and Banerdt, W. B. (2022). Newly formed craters on Mars located using seismic and acoustic wave data from InSight. *Nature Geoscience*, 15.

Gendre, H., Badro, J., Wehr, N., and Borensztajn, S. (2022). Martian core composition from experimental high-pressure metal-silicate phase equilibria. *Geochemical Perspectives Letters*, 21.

Haney, M. M., Fee, D., McKee, K. F., Lyons, J. J., Matoza, R. S., Wech, A. G., Tepp, G., Searcy, C., and Mikesell, T. D. (2020). Co-eruptive tremor from Bogoslof volcano: seismic wavefield composition at regional distances. *Bulletin of Volcanology*, 82(2).

Huang, D., Badro, J., Brodholt, J., and Li, Y. (2019). Ab Initio Molecular Dynamics Investigation of Molten Fe–Si–O in Earth's Core. *Geophysical Research Letters*, 46(12):6397–6405.

InSight Mars SEIS Data Service (2019). Seis raw data, insight mission.

InSight Marsquake Service (2023). Mars seismic catalogue, insight mission; v13 2023-01-01.

Irving, J., Lekić, V., Duran, C., Drilleau, M., Kim, D., Rivoldini, A., Khan, A., Samuel, H., Antonangeli, D., Banerdt, W. B., Beghein, C., Bozdag, E., Ceylan, S., Clinton, J., Garcia, R., Giardini, D., Horleston, A., Huang, Q., Hurst, K. J., Kawamura, T., King, S., Knapmeyer, M., Li, J., Lognonné, P., Maguire, R., Panning, M. P., Plesa, A., Schimmel, M., Schmerr, N. C., Stähler, S., Stutzmann, E., and Xu, Z. (2023). First observations of core-transiting seismic phases on mars. *Proc. Nat. Acad. Sci.*, 120(18):e2217090120. doi 10.1073/pnas.221709012.

Jacob, A., Plasman, M., Perrin, C., Fuji, N., Lognonné, P., Xu, Z., Drilleau, M., Brinkman, N., Stähler, S., Sainton, G., Lucas, A., Giardini, D., Kawamura, T., Clinton, J., and Banerdt, W. (2022). Seismic sources of insight marsquakes and seismotectonic context of elysium planitia, mars. *Tectonophysics*, 837:229434.

Jing, Z. and ichiro Karato, S. (2008). Compositional effect on the pressure derivatives of bulk modulus of silicate melts. *Earth and Planetary Science Letters*, 272(1-2):429–436.

Karato, S.-I. and Wu, P. (1993). Rheology of the upper mantle: A synthesis. *Science*, 260:771–778.

Kawaguchi, S. I., Nakajima, Y., Hirose, K., Komabayashi, T., Ozawa, H., Tateno, S., Kuwayama, Y., Tsutsui, S., and Baron, A. Q. R. (2017). Sound velocity of liquid Fe-Ni-S at high pressure. *Journal of Geophysical Research: Solid Earth*, 122(5):3624–3634.

Kawamura, T., Clinton, J., Zenhäusern, G., Ceylan, S., and Horleston, A. (2022). Largest Marsquake Ever Detected by InSight: S1222a. *submitted to Geophys. Res. Lett.*

Kim, D., Banerdt, W. B., Ceylan, S., Giardini, D., Beghein, C., Carrasco, S., Charalambous, C., Clinton, J., Drilleau, M., Golombek, M., Joshi, R., Khan, A., Li, J., Maguire, R., Pike, W. T., Samuel, H., Schimmel, M., Schmerr, N. C., Stutzmann, E., Wieczorek, M., Xu, Z., Batov, A., Bozdag, E., Dahmen, N., Davis, P., Gudkova, T., Horleston, A., Huang, Q., Kawamura, T., King, S. D., Mclennan, S. M., Nimmo, F., Plasman, M., Plesa, A. C., Stepanova, I. E., Weidner, E., Daubar, I. J., Fernando, B., Garcia, R. F., Posiolova, L. V., and Panning, M. P. (2022). Surface waves and crustal structure on Mars. *Science*, 421:417–421.

Knapmeyer-Endrun, B., Panning, M. P., Bissig, F., Joshi, R., Khan, A., Kim, D., Lekić, V., Tauzin, B., Tharimena, S., Plasman, M., Compaire, N., Garcia, R. F., Margerin, L., Schimmel, M., Éléonore Stutzmann, Schmerr, N., Bozdağ, E., Plesa, A.-C., Wieczorek, M. A., Broquet, A., Antonangeli, D., McLennan, S. M., Samuel, H., Michaut, C., Pan, L., Smrekar, S. E., Johnson, C. L., Brinkman, N., Mittelholz, A., Rivoldini, A., Davis, P. M., Lognonné, P., Pinot, B., Scholz, J.-R., Stähler, S., Knapmeyer, M., van Driel, M., Giardini, D., and Banerdt, W. B. (2021). Thickness and structure of the martian crust from insight seismic data. *Science*, 373(6553):438–443.

Komabayashi, T. (2014). Thermodynamics of melting relations in the system Fe-FeO at high pressure: Implications for oxygen in the Earth's core. *Journal of Geophysical Research: Solid Earth*, page 2014JB010980.

Konopliv, A. S., Park, R. S., and Folkner, W. M. (2016). An improved JPL Mars gravity field and orientation from Mars orbiter and lander tracking data. *Icarus*, 274:253–260.

Konopliv, A. S., Park, R. S., Rivoldini, A., Baland, R. M., Le Maistre, S., Van Hoolst, T., Yseboodt, M., and Dehant, V. (2020). Detection of the Chandler Wobble of Mars From Orbiting Spacecraft. *Geophysical Research Letters*, 47(21):1–9.

Le Maistre, S., Rivoldini, A., Caldiero, A., Yseboot, M., Baland, M.-R., Beuthe, M., Van Hoolst, T. ., Dehant, V., Folkner, W. M., Buccino, D., Kahan, D., Marty, J.-C., Antonangeli, D., Badro, J., Drilleau, M., Konopliv, A., Péters, M.-J., Plesa, A.-C., Samuuel, H., Tosi, N., Wieczorek, M., Lognonné, P., Panning, M., Smrekar, S., and Banerdt, W. B. (2023). Spin state and deep interior structure of mars from insight radio tracking. *Nature*, page Accepted.

Matsui, M. (1996). Molecular dynamics simulation of structures, bulk moduli, and volume thermal expansivities of silicate liquids in the system CaO-MgO-Al2O3-SiO2. *Geophysical Research Letters*, 23(4):395–398.

Morard, G., Antonangeli, D., Bouchet, J., Rivoldini, A., Boccato, S., Miozzi, F., Boulard, E., Bureau, H., Mezouar, M., Prescher, C., Chariton, S., and Greenberg, E. (2022). Structural and Electronic Transitions in Liquid FeO Under High Pressure. *Journal of Geophysical Research: Solid Earth*, 127(11):e2022JB025117.

Morard, G., Nakajima, Y., Andrault, D., Antonangeli, D., Auzende, A. L., Boulard, E., Cervera, S., Clark, A. N., Lord, O. T., Siebert, J., Svitlyk, V., Garbarino, G., and Mezouar, M. (2017). Structure and Density of Fe-C Liquid Alloys Under High Pressure. *Journal of Geophysical Research: Solid Earth*, 122(10):7813–7823.

Morard, G., Siebert, J., Andrault, D., Guignot, N., Garbarino, G., Guyot, F., and Antonangeli, D. (2013). The Earth's core composition from high pressure density measurements of liquid iron alloys. *Earth and Planetary Science Letters*, 373(0):169–178.

Nishida, K., Shibazaki, Y., Terasaki, H., Higo, Y., Suzuki, A., Funamori, N., and Hirose, K. (2020). Effect of sulfur on sound velocity of liquid iron under Martian core conditions. *Nature Communications*, 11(1):1954.

Nissen-Meyer, T., van Driel, M., Stähler, S. C., Hosseini, K., Hempel, S., Auer, L., Colombi, A., and Fournier, A. (2014). Axisem: broadband 3-d seismic wavefields in axisymmetric media. *Solid Earth*, 5(1):425–445.

Peyre, G. (2009). Toolbox fast marching. *MATLAB Central File Exchange Select*, 2.

Piet, H., Badro, J., and Gillet, P. (2017). Geochemical Constraints on the Size of the Moon-Forming Giant Impact. *Geophysical Research Letters*, 44(23):11,770–11,777.

Posiolova, L. V., Lognonné, P., Banerdt, W. B., Clinton, J., Collins, G. S., Kawamura, T., Ceylan, S., Daubar, I. J., Fernando, B., Froment, M., Giardini, D., Malin, M. C., Miljković, K., Stähler, S. C., Xu, Z., Banks, M. E., Beucler, E., Cantor, B. A., Charalambous, C., Dahmen, N., Davis, P., Drilleau, M., Dundas, C. M., Durán, C., Euchner, F., Garcia, R. F., Golombek, M., Horleston, A., Keegan, C., Khan, A., Kim, D., Larmat, C., Lorenz, R., Margerin, L., Menina, S., Panning, M., Pardo, C., Perrin, C., Pike, W. T., Plasman, M., Rajšić, A., Rolland, L., Rougier, E., Speth, G., Spiga, A., Stott, A., Susko, D., Teanby, N. A., Valeh, A., Werynski, A., Wójcicka, N., and Zenhäusern, G. (2022). Largest recent impact craters on mars: Orbital imaging and surface seismic co-investigation. *Science*, 378(6618):412–417.

Ranalli, G. (1995). *Rheology of the Earth, 2nd edn.* Chapman and Hall, London.

Ruedas, T. and Breuer, D. (2017). On the relative importance of thermal and chemical buoyancy in regular and impact-induced melting in a Mars-like planet. *Journal of Geophysical Research: Planets*, 122(7):1554–1579.

Samuel, H., Ballmer, M. D., Padovan, S., Tosi, N., Rivoldini, A., and Plesa, A.-C. (2021). The thermo-chemical evolution of mars with a strongly stratified mantle. *Journal of Geophysical Research: Planets*, 126(4):e2020JE006613. e2020JE006613 2020JE006613.

Samuel, H., Drilleau, M., Garcia, R. F., Rivoldini, A., Lognonné, P., Huang, Q., and Banerdt, W. B. (2022). Testing the presence deep martian mantle layering in the light of insight seismic data. In *EPSC meeting, Granada, Spain*.

Samuel, H., Lognonné, P. H., Panning, M., and Lainey, V. (2019). The rheology and thermal history of mars revealed by the orbital evolution of Phobos. *Nature*, 569:523–527. doi:10.1038/s41586-019-1202-7.

Sanloup, C., Jambon, A., and Gillet, P. (1999). A simple chondritic model of Mars. *Physics of the Earth and Planetary Interiors*, 112(1-2):43–54.

Sethian, J. A. and Popovici, A. M. (1999). 3-d traveltime computation using the fast marching method. *Geophysics*, 64(2):516–523.

Shimoyama, Y., Terasaki, H., Urakawa, S., Takubo, Y., Kuwabara, S., Kishimoto, S., Watanuki, T., Machida, A., Katayama, Y., and Kondo, T. (2016). Thermoelastic properties of liquid Fe-C revealed by sound velocity and density measurements at high pressure. *Journal of Geophysical Research: Solid Earth*, 121(11):7984–7995.

Stähler, S. C., Khan, A., Banerdt, W. B., Lognonné, P., Giardini, D., Ceylan, S., Drilleau, M., Duran, A. C., Garcia, R. F., Huang, Q., Kim, D., Lekic, V., Samuel, H., Schimmel, M., Schmerr, N., Sollberger, D., Stutzmann, É., Xu, Z., Antonangeli, D., Charalambous, C., Davis, P. M., Irving, J. C. E., Kawamura, T., Knapmeyer, M., Maguire, R., Marusiak, A. G., Panning, M. P., Perrin, C., Plesa, A.-C., Rivoldini, A., Schmelzbach, C., Zenhäusern, G., Beucler, É., Clinton, J., Dahmen, N., van Driel, M., Gudkova, T., Horleston, A., Pike, W. T., Plasman, M., and Smrekar, S. E. (2021). Seismic detection of the martian core. *Science*, 373(6553):443–448.

Steenstra, E. S. and van Westrenen, W. (2018). A synthesis of geochemical constraints on the inventory of light elements in the core of Mars. *Icarus*, 315:69–78.

Steenstra, S., Eising, T., Webers, B., Klemme, M., and van Westrenen, W. (2018). Evidence for a sulfur-undersaturated lunar interior from the solubility of sulfur in lunar melts and sulfide-silicate partitioning of siderophile elements. *Geochimica et Cosmochimica Acta*, 231:130–156.

Tagawa, S., Gomi, H., Hirose, K., and Ohishi, Y. (2022a). High-Temperature Equation of State of FeH: Implications for Hydrogen in Earth's Inner Core. *Geophysical Research Letters*, 49(5):e2021GL096260.

Tagawa, S., Helffrich, G., Hirose, K., and Ohishi, Y. (2022b). High-Pressure Melting Curve of FeH: Implications for Eutectic Melting Between Fe and Non-Magnetic FeH. *Journal of Geophysical Research: Solid Earth*, 127(6):e2022JB024365.

Terasaki, H., Nishida, K., Shibazaki, Y., Sakamaki, T., Suzuki, A., Ohtani, E., and Kikegawa, T. (2010). Density measurement of Fe3C liquid using X-ray absorption image up to 10 GPa and effect of light elements on compressibility of liquid iron. *J. Geophys. Res.*, 115(B6).

Tsuno, K., Grewal, D. S., and Dasgupta, R. (2018). Core-mantle fractionation of carbon in Earth and Mars: The effects of sulfur. *Geochimica et Cosmochimica Acta*, 238:477–495.

Umemoto, K. and Hirose, K. (2020). Chemical compositions of the outer core examined by first principles calculations. *Earth and Planetary Science Letters*, 531:116009.

Wade, J. and Wood, B. (2005). Core formation and the oxidation state of the Earth. *Earth and Planetary Science Letters*, 236(1-2):78–95.

Wieczorek, M. A., Broquet, A., McLennan, S. M., Rivoldini, A., Golombek, M., Antonangeli, D., Beghein, C., Giardini, D., Gudkova, T., Gyalay, S., Johnson, C. L., Joshi, R., Kim, D., King, S. D., Knapmeyer-Endrun, B., Lognonné, P., Michaut, C., Mittelholz, A., Nimmo, F., Ojha, L., Panning, M. P., Plesa, A.-C., Siegler, M. A., Smrekar, S. E., Spohn, T., and Banerdt, W. B. (2022). Insight constraints on the global character of the martian crust. *Journal of Geophysical Research: Planets*, 127(5):e2022JE007298. e2022JE007298 2022JE007298.

Xu, F., Morard, G., Guignot, N., Rivoldini, A., Manthilake, G., Chantel, J., Xie, L., Yoneda, A., King, A., Boulard, E., Pandolfi, S., Ryerson, F. J., and Antonangeli, D. (2021). Thermal expansion of liquid Fe-S alloy at high pressure. *Earth and Planetary Science Letters*, 563:116884.

Yoder, C. F., Konopliv, A. S., Yuan, D. N., Standish, E. M., and Folkner, W. M. (2003). Fluid Core Size of Mars from Detection of the Solar Tide. *Science*, 300:299–303.

Yoshizaki, T. and McDonough, W. F. (2020). The composition of Mars. *Geochimica et Cosmochimica Acta*, 273:137–162.

Young, E. D., Shahar, A., and Schlichting, H. E. (2023). Earth shaped by primordial H2 atmospheres. *Nature*, 616(7956):306–311.
